# From invitation to eviction: How plants control arbuscular mycorrhizal symbiosis

**DOI:** 10.1080/15592324.2026.2733232

**Published:** 2026-09-16

**Authors:** Marvi Korai, Shakal Khan Korai, Shahrukh Khan, Shengnan Li, Usman Zulfiqar, Mohammed S. Alotaibi, Sokhibjon Abdusamatov, Anvar Rakhmatov, Mohd Asif Shah, Ying Sun

**Affiliations:** a College of Animal Science and Technology, Yangzhou University, Yangzhou, People's Republic of China; b Department of Agronomy, Faculty of Agriculture and Environment, The Islamia University of Bahawalpur, Bahawalpur, Pakistan; c Department of Biology, Nakhchivan State University, Nakhchivan, Azerbaijan; d Department of Biology, Turabah University College, Taif University, Taif, Saudi Arabia; e Department of Microbiology and Biotechnology, National University of Uzbekistan, Tashkent, Uzbekistan; f Research Institute of Vegetable, Melon Crops and Potato, Tashkent District, Tashkent Region, Uzbekistan; g Kardan University, Parwane Du, Kabul, Afghanistan

**Keywords:** Arbuscular mycorrhizal symbiosis, plant signaling, nutrient exchange, arbuscule turnover, fungal effectors

## Abstract

Arbuscular mycorrhizal (AM) symbiosis is often presented as a linear sequence of fungal recognition, root colonization, arbuscule formation, and nutrient exchange. This view underrepresents the repeated regulatory transitions through which plants influence symbiotic establishment, function, and persistence. Here, we propose a checkpoint framework organized around invitation, admission, accommodation, investment, maintenance, and termination and renewal. We examine how nutrient status, carbon availability, hormonal and immune signaling, systemic root–shoot communication, and environmental context regulate fungal recruitment, intracellular entry, interface construction, resource exchange, and arbuscule turnover. We distinguish strong mechanistic evidence from correlative observations and emphasize that gene expression, colonization abundance, or arbuscule degeneration alone do not demonstrate resource flux, performance-sensitive evaluation, partner-level sanction, or selective interface termination. Evidence is strongest for plant control of presymbiotic signaling, cellular accommodation, interface construction and resourcing, nutrient acquisition, and regulated arbuscule turnover. By contrast, direct performance-sensitive evaluation of individual fungal interfaces remains insufficiently demonstrated. AM fungi also providing regulatory inputs: fungal signals, secreted molecules, and small RNAs can modify host processes, while fungal physiology and extraradical networks influence nutrient acquisition and allocation. Plant control is therefore substantial but not absolute, operating through asymmetrical, reciprocal regulation across interface, whole-plant, and fungal-network scales. We conclude by outlining experiments that link fungal nutrient contribution, plant response or allocation, and subsequent fate at the same interface, with fungal fitness additionally required for claims of partner-level sanction. Such experiments provide tests for distinguishing developmental and physiological regulation from performance-sensitive interface maintenance.

## Graphical abstract

**Figure:**
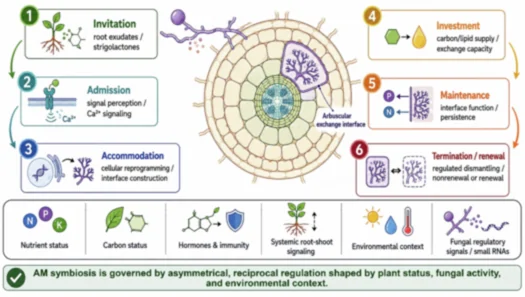

## 1. Introduction

Arbuscular mycorrhizal (AM) symbiosis is frequently introduced as a mutualistic association in which fungi improve plant access to mineral nutrients in exchange for plant-derived carbon.^1^ ^,^ ^2^ Although accurate, this description can obscure the central regulatory problem faced by the plant. Supporting an intracellular fungal symbiont requires extensive cellular reprogramming, construction of a specialized exchange membrane, and sustained allocation of sugars and lipids.^3^ ^,^ ^4^ Plants must therefore regulate not only whether AM fungi reach and enter roots, but also where intracellular accommodation occurs, how resources are allocated to symbiotic interfaces, and how long those interfaces are maintained. AM symbiosis being consequently better viewed as a dynamically regulated association than as a developmental program that becomes autonomous after initial recognition.

AM fungi colonizing the root cortex and form highly branched intracellular structures known as arbuscules. Each arbuscule remains separated from the plant cytoplasm by the plant-derived periarbuscular membrane, creating an extensive interface across which fungal phosphate and other mineral nutrients can be transferred to the host. In return, the plant supplies the fungus with reduced carbon, including carbohydrates and host-derived lipids.^1^ ^,^ ^5-7^ The outcome of this exchange, however, is context dependent. Plant responses to colonization vary with nutrient availability, host genotype, fungal taxon or isolate, carbon status, developmental stage, and environmental conditions, and the association can range from strongly beneficial to weak, neutral, or costly from the plant perspective.^8^ Reciprocal-allocation experiments further indicate that resource exchange can respond to partner contribution, although the mechanisms and spatial scales at which such responses operate remain unresolved.^9^

Plant regulation begins before physical contact. Nutrient-responsive changes in root exudation influence fungal metabolism and growth, with strigolactones providing a well-established example of plant-derived compounds that stimulate AM fungal hyphal branching and increase the probability of root encounter.^10^ Fungal chitooligosaccharides and lipochitooligosaccharides can subsequently activate plant symbiotic signaling, but recognition alone does not ensure successful colonization.^11^ Entry requires coordinated calcium signaling, transcriptional reprogramming, cytoskeletal rearrangement, membrane trafficking, and formation of an intracellular accommodation pathway. Thus, plants actively create the cellular conditions required for fungal entry and arbuscule development rather than merely recognizing and tolerating fungal presence.

Regulation also continues after entry. Plants construct the periarbuscular membrane, target symbiosis-specific transporters to defined membrane domains, and activate carbon and lipid supply pathways that sustain fungal development. Genetic disruption of these processes can permit initial colonization while impairing arbuscule branching, nutrient exchange, or persistence. For example, loss of the symbiotic phosphate transporter PT4 in *Medicago truncatula* disrupts normal arbuscule maintenance, linking plant nutrient-import machinery to interface lifespan.^2^ Transcriptional feedback circuits and ARK-family receptor kinases provide additional evidence that arbuscule development and persistence remain subject to active host regulation.^12^ ^,^ ^13^ At the same time, colonized roots are spatially and developmentally heterogeneous: neighboring cortical cells may be uncolonized, preparing for entry, supporting developing or mature arbuscules, or dismantling degenerating interfaces. Spatial co-transcriptomic analyzes resolve distinct plant and fungal states across these stages,^14^ while live analyzes of phosphate-transporter dynamics reveal heterogeneity in transporter behavior and arbuscule developmental trajectories.^15^ Consequently, whole-root colonization percentages alone cannot define the activity, nutritional contribution, or fate of individual interfaces.

Plant regulation is nevertheless only one side of the interaction. AM fungi being not passive recipients of host regulation; fungal signals, secreted effectors, CLE-like peptides, small RNAs, and other transported molecules can modify plant signaling, immunity, accommodation, and symbiotic development. For example, an AM fungal CLE-like peptide can engage a plant regulatory pathway, illustrating that fungi may modify processes otherwise interpreted primarily as components of host control.^16^ Plant regulation is therefore substantial but not unilateral and operates within an asymmetrical, reciprocal interaction shaped by fungal activity, plant physiological status, resource availability, and environmental context. Mechanisms demonstrated in one host–fungus combination are not assumed here to be conserved across all AM associations; throughout the review, broadly shared features are distinguished from model-specific mechanisms and hypotheses requiring cross-system testing.

**Figure 1.**
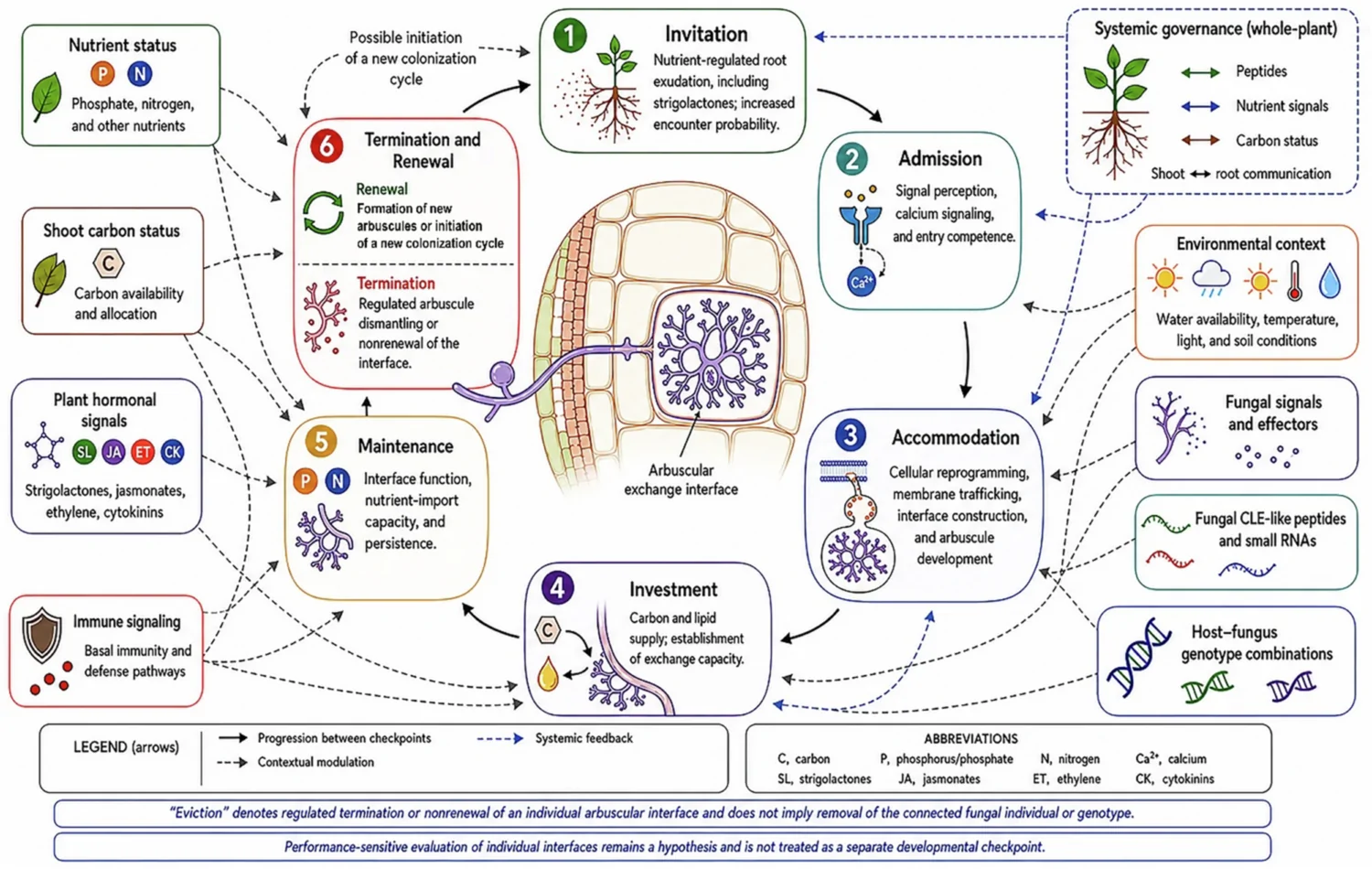
A six-checkpoint model of plant regulation in arbuscular mycorrhizal symbiosis. The figure summarizes published evidence for six overlapping checkpoints in AM symbiosis: invitation, admission, accommodation, investment, maintenance, and termination/renewal. These checkpoints are influenced by nutrient and carbon status, hormonal and immune signaling, systemic whole-plant regulation, environmental context, fungal regulatory molecules, and host–fungus genotype combinations. “Eviction” is used here as shorthand for regulated termination or nonrenewal of an individual arbuscular interface and does not imply removal of the connected fungal individual or genotype. Performance-sensitive evaluation of individual interfaces remains a hypothesis and is therefore not treated as a separate checkpoint.

In this review, we organize plant regulation of AM symbiosis into six overlapping checkpoints: invitation, admission, accommodation, investment, maintenance, and termination and renewal. These checkpoints describe biological transitions rather than a strictly linear sequence, and they operate at different spatial and developmental scales. Performance-sensitive evaluation is not treated as a separate checkpoint but as a testable hypothesis that could influence the maintenance or termination of established interfaces. Demonstrating such regulation would require measured fungal contribution to be linked to a differential plant response and the subsequent fate of the same interface. We use the term “control” to describe plant processes that alter the probability, location, extent, activity, or duration of fungal colonization, rather than complete or unilateral command over the fungal partner. Terms such as “invitation” and “eviction” are used as operational metaphors for measurable regulatory processes and do not imply cognition or intention. In particular, “eviction” refers to regulated termination or nonrenewal of an individual arbuscular interface and does not imply removal of the connected fungal individual or genotype. Figure 1 and Table 1 summarize published evidence supporting the six-checkpoint framework, whereas Table 2 defines the operational terminology and evidentiary requirements used throughout this review. By examining AM symbiosis from invitation to eviction, we distinguish well-supported regulatory mechanisms from interpretations that remain consistent with, but not yet demonstrated by, available evidence.

**Table 1.** Checkpoint framework for regulation of arbuscular mycorrhizal symbiosis and the evidence supporting each process.

Checkpoint or regulatory layer	Regulated process	Representative regulators or mechanisms	Representative evidence	Supported interpretation	Important limitation	References
Invitation	Rhizosphere signaling that increases fungal activity and the probability of root encounter	PHR-dependent nutrient signaling; strigolactone biosynthesis and export; NOPE1-dependent exudate activity	Genetic perturbation of signal production, export, or exudate function alters fungal presymbiotic responses, hyphopodium formation, or colonization	Plants regulate conditions that make fungal encounter more probable	Fungal activation or branching does not demonstrate partner-specific recruitment or successful entry	[10,17-20]
Admission	Conversion of fungal perception into a host-cell state permissive for intracellular entry	COs/LCOs; LysM receptor complexes including CERK1 and LYR proteins; nuclear calcium signaling; CCaMK	Purified fungal signals activate symbiotic signaling, whereas receptor or CCaMK perturbation alters signal responses and AM establishment	Signal perception must be coupled to downstream cellular competence for entry	No single signal or receptor uniquely identifies all AM fungi; calcium signaling does not guarantee entry	[11,21-24]
Accommodation	Host-cell reorganization and construction and differentiation of the intracellular interface	CCaMK–CYCLOPS–DELLA–RAM1 signaling; prepenetration apparatus; VAPYRIN; EXO70I; SYP13IIα-dependent secretion	Cellular imaging and genetic perturbations demonstrate active host preparation for penetration, arbuscule branching, and specialized interface formation	Plants actively reorganize cortical cells and construct much of the host-derived interface	Fungal branching also shapes interface geometry, and interface construction does not itself demonstrate resource exchange	[3,4,25-27]
Investment	Host carbon and lipid provision and establishment of transport and electrochemical capacity for exchange	RAM1–WRI regulation; FatM/RAM2-dependent lipid provision; PT4; HA1	Isotope and mutant studies demonstrate plant-to-fungus lipid transfer, while PT4 and HA1 perturbations impair symbiotic phosphate acquisition	Plants provide resources and establish major components of the exchange machinery	Expression or localization establishes potential capacity, not net flux; transporter phenotypes do not identify the variable sensed	[1,2,7,28,29]
Maintenance	Persistence and physiological state of established arbuscular interfaces	PT4; PT11 dynamics; WRI5a–ERM1–ERF12 feedback; ARK-family signaling; nutrient-sensitive maintenance	Transporter perturbations alter arbuscule lifespan; live imaging reveals heterogeneous interface trajectories; ARK loss impairs sustained symbiosis and fungal life-cycle progression	Established interfaces remain subject to host nutritional and signaling regulation	Variation in transporter abundance or lifespan does not demonstrate sensing of fungal contribution or performance-sensitive evaluation	[2,12,15,30,31]
Termination and renewal	Regulated dismantling or nonrenewal of established interfaces and subsequent formation of new interfaces	MYB1; DELLA/NSP1; degeneration-associated hydrolases; membrane and transporter turnover	MYB1 perturbation alters the host degeneration program, while live analyzes resolve interface collapse and turnover	Plants actively participate in arbuscule dismantling and interface turnover	Arbuscule degeneration does not demonstrate performance-sensitive eviction or sanction, and local interface loss does not eliminate the connected fungal genotype	[2,15,32,33]
Systemic governance (cross-cutting)	Whole-plant coordination of checkpoint activity according to nutrient status, shoot signals, and existing colonization	CLE53–SUNN; CEP–CRA2; HY5-dependent shoot-to-root signaling; systemic phosphate regulation	Split-root, grafting, and genetic studies demonstrate regulation beyond individual colonization sites; phosphate repletion preferentially suppresses new interface development	Systemic signals modify root-wide opportunities for colonization and renewal	Systemic regulation does not establish differential fate of individual mature interfaces, and several mobile signals remain unresolved	[34-38]
Fungal regulatory inputs (cross-cutting)	Fungal modulation of host regulatory processes and network-scale resource allocation	SP7; RiNLE1; RiCLE1; fungal small RNAs; fungal nutrient allocation	Fungal molecules interact with host regulatory components; functional cross-kingdom small-RNA transfer has been demonstrated; isotope studies show resource-responsive fungal allocation	Plant checkpoints operate within asymmetrical, reciprocal plant–fungus regulation	Native delivery or fungal loss-of-function evidence remains incomplete for several effectors, and mechanisms demonstrated in one host–fungus combination cannot be assumed to be conserved	[16,39-42]

Note: Invitation, admission, accommodation, investment, maintenance, and termination and renewal are the six core checkpoints used in this review. Systemic governance and fungal regulatory inputs are cross-cutting regulatory layers rather than additional checkpoints. Performance-sensitive evaluation is treated as a hypothesis concerning established interfaces, particularly maintenance or termination, and not as a separate developmental checkpoint. References are representative primary studies selected for direct support and are not intended to be exhaustive.

**Table 2.** Operational definitions and evidentiary requirements for terms used to describe regulation of arbuscular mycorrhizal symbiosis.

Term	Operational meaning in this review	Minimum evidence required	Evidence that does not establish the term by itself	Recommended cautious wording	References
Control	A plant-derived process causally alters the probability, location, extent, activity, or duration of a defined component of fungal colonization or symbiotic function.	Functional perturbation of a plant process linked to a defined symbiotic outcome at an identified developmental or spatial scale.	Differential gene expression; correlation with colonization; changes in plant biomass alone.	“The plant process regulates…” or “is required for…,” with the affected process and scale specified.	[2,33,35]
Invitation	Plant regulation of signals that increase fungal activation or the probability of root encounter.	Signal production or export, a reproducible fungal response, and an effect on subsequent symbiotic establishment.	Hyphal branching in response to an isolated compound alone.	“Increases encounter probability” or “promotes presymbiotic fungal activation.”	[10,17-20]
Admission	Conversion of fungal perception into a host-cell state permissive for epidermal or cortical entry.	Receptor or signaling perturbation that alters entry after fungal contact, with preceding signaling responses measured where possible.	Ligand perception, calcium oscillations, or receptor binding without measurement of entry.	“Establishes competence for entry” or “promotes intracellular entry.”	[11,21-24]
Accommodation	Host-cell reorganization and construction of the intracellular interface required for fungal passage and arbuscule development.	Cell-resolved evidence connecting host signaling, transcription, trafficking, or structural remodeling to intracellular fungal development.	Increased total colonization or expression of a symbiosis marker.	“Supports intracellular accommodation” or “contributes to interface construction.”	[3,4,25-27]
Investment	Host carbon and lipid provision and establishment of transport and electrochemical capacity for resource exchange.	Direct resource tracing or functional perturbation of a defined biosynthetic or transport pathway linked to resource provision or acquisition.	Induction or localization of a candidate sugar, lipid, or nutrient transporter alone.	“Contributes to host resource provision” or “establishes exchange capacity.”	[1,2,7,28,29]
Maintenance	Regulation of the persistence and functional state of an established arbuscular interface.	Evidence that a defined plant process alters persistence, physiological state, or lifespan of an established interface.	Transporter abundance, presence of a mature arbuscule, or altered total colonization without evidence concerning persistence of established interfaces.	“Contributes to interface maintenance” or “regulates arbuscule persistence.”	[2,15,30,31]
Termination and renewal	Regulated dismantling or nonrenewal of an established arbuscular interface, with subsequent formation of new interfaces treated as a distinct renewal process.	Temporal, molecular, or genetic evidence linking a defined process to interface dismantling, nonrenewal, or formation of subsequent interfaces.	Collapsed arbuscules at a single endpoint or reduced total colonization without developmental resolution.	“Regulated interface termination,” “arbuscule turnover,” or “renewal,” according to the process measured.	[15,32,33,38]
Evaluation	A plant response varies with a measured fungal functional contribution at a defined biological scale.	Measured fungal contribution and a differential plant response under comparable conditions. For performance-sensitive interface maintenance, fungal contribution, plant response or allocation, and subsequent fate must be linked at the same interface.	Variation in transporter abundance, arbuscule morphology or lifespan, total colonization, or resource allocation measured at a different spatial scale.	“Is consistent with performance-sensitive regulation” unless contribution and response are linked directly.	[9,15,43]
Reward	Greater plant resource allocation to a distinguishable partner, fungal compartment, or root sector associated with greater fungal contribution.	Directly measured differential plant allocation associated with measured differences in fungal contribution under comparable conditions.	Greater plant carbon transfer after a general change in nutrient availability, or greater colonization without direct resource measurements.	Prefer “preferential allocation” unless the biological unit and resource transfer are directly resolved.	[9,43]
Restriction	Reduction in new entry events, colonization spread, arbuscule formation, or interface renewal.	Temporal or developmental evidence showing where further colonization is limited.	Lower colonization at a single endpoint.	“Restricts new colonization” or “reduces interface renewal.”	[35,36,38,44]
Turnover	Regulated developmental dismantling of an individual arbuscule and its associated host interface; subsequent renewal is a separate event.	Time-resolved morphological or molecular evidence of arbuscule degeneration and interface recycling.	Presence of collapsed arbuscules in fixed tissue alone.	“Regulated arbuscule turnover.”	[15,32,33]
Sanction	Contribution-dependent host treatment that imposes a negative fitness consequence on a lower-contributing fungal partner.	Measured variation in fungal contribution, contribution-dependent differential host treatment, and a resulting reduction in fungal growth or reproductive fitness.	Premature arbuscule degeneration in a plant-transporter mutant; reduced colonization under nutrient sufficiency; preferential allocation without a demonstrated fungal fitness consequence.	Use “preferential allocation” or “restriction” unless the full sanction criteria are satisfied.	[9,43]
Eviction	Conceptual shorthand for regulated termination or nonrenewal of an individual arbuscular interface.	Evidence of active plant participation in interface termination or regulated withdrawal of support; performance-sensitive eviction additionally requires fungal contribution to be linked to the subsequent fate of that same interface.	Normal arbuscule senescence, degeneration in a transport mutant, or reduction in total fungal abundance.	Prefer “regulated interface termination”; reserve “eviction” for explicitly defined conceptual use, not removal of the connected fungal individual or genotype.	[2,15,33]
Partner choice	Differential establishment or support among simultaneously available, distinguishable fungal partners, linked to partner traits or functional contribution.	Simultaneous availability of distinguishable fungal partners under comparable conditions, differential establishment or host support, and evidence linking that difference to partner contribution or host outcome.	Different colonization levels in separate single-fungus experiments.	“Partner-dependent compatibility” or “preferential association” unless simultaneous, contribution-linked differential treatment is demonstrated.	[9,43,45]
Symbiotic memory	A previous condition causes a persistent change in a later AM response after the initiating condition and its direct physiological effects have ended.	A history-dependent design under matched current conditions, ideally with perturbation of a persistent causal state that alters the later response.	Differences between continuously treated and untreated plants, or persistent phenotypic differences explained by residual nutrient status, root architecture, fungal abundance, or other current physiological differences.	“History-dependent response” until a persistent mechanism is established.	[46,47]

Note: Invitation, admission, accommodation, investment, maintenance, and termination and renewal are the six core checkpoints defined in Table 1 and used consistently throughout this review. Evaluation, reward, restriction, turnover, sanction, eviction, partner choice, and symbiotic memory describe particular processes or interpretations and are not additional checkpoints. Systemic governance and fungal regulatory inputs are cross-cutting regulatory layers rather than additional checkpoints. Performance-sensitive evaluation is a hypothesis concerning established interfaces, particularly maintenance or termination, rather than an established developmental checkpoint. The cited studies provide the empirical or conceptual basis for these definitions but do not necessarily satisfy the minimum evidentiary criteria specified here. Terms such as “reward,” “sanction,” “choice,” “eviction,” and “memory” are operational shorthand and do not imply cognition or intention.

## 2. Review methodology

The literature reviewed was obtained by searching major scientific databases (PubMed, Web of Science, and Google Scholar) using combinations and synonyms of the keywords: arbuscular mycorrhizal symbiosis, plant regulation, strigolactone, fungal recognition, symbiotic signaling, intracellular accommodation, periarbuscular membrane, carbon and lipid transfer, nutrient exchange, arbuscule maintenance, arbuscule turnover, autoregulation of mycorrhization, fungal effectors, small RNAs, partner choice, and sanction. The publications included in this review ranged from 1997 to 2026.

The review focused mainly on studies addressing fungal recruitment and entry, intracellular accommodation, interface construction and resource exchange, arbuscule maintenance and termination, systemic regulation, and fungal regulatory effects on host processes. Greater weight was given to peer-reviewed primary studies providing genetic or physiological perturbation, direct resource-transfer measurements, spatial or time-resolved analyzes, or clearly defined symbiotic phenotypes. Studies were interpreted according to the host–fungus system, biological scale, perturbation, and measured outcome. Review articles were used mainly to identify foundational literature and provide broader conceptual context, while studies lacking sufficient methodological or mechanistic detail were not used as primary support for specific mechanistic claims.

## 3. The checkpoint framework and evidence criteria

Describing plants as controlling AM symbiosis does not imply that the host commands every aspect of fungal behavior or determines the outcome independently of its partner. Rather, plant control refers to host-derived processes that alter the probability, location, extent, activity, or duration of fungal colonization. Plants regulate rhizosphere signaling, fungal entry, intracellular accommodation, construction of the periarbuscular interface, resource provision and nutrient uptake, and arbuscule maintenance and termination. Fungal growth, nutrient acquisition and allocation, and regulatory molecules simultaneously influence these processes, so plant control remains conditional rather than unilateral.^5^ ^,^ ^48^ Plant control is therefore conditional and distributed within an interdependent association.

The checkpoint framework differs from a conventional description of AM development as a sequence of morphological stages. A developmental stage describes the current state of the interaction, whereas a checkpoint identifies a transition whose progression depends on plant and fungal conditions. We distinguish six core, overlapping checkpoints: invitation, admission, accommodation, investment, maintenance, and termination and renewal. Invitation concerns whether plant-derived signals increase the likelihood of fungal contact; admission concerns whether fungal perception leads to entry; accommodation encompasses the cellular reorganization required for intracellular fungal development; investment concerns plant carbon and lipid provision and establishment of the transport capacity required for exchange; maintenance concerns continued support of a functional arbuscule; and termination and renewal encompass regulated dismantling or nonrenewal of established interfaces and subsequent formation of new interfaces or colonization events. These checkpoints are neither strictly sequential nor equivalent in scale, and different checkpoints can operate simultaneously in neighboring cells of the same root.^14^ ^,^ ^48^ ^,^ ^49^ Figure 1 and Table 1 summarize published evidence supporting regulation across these six checkpoints.

Performance-sensitive evaluation is treated here not as an established seventh checkpoint, but as a hypothesis concerning whether fungal contribution influences maintenance or termination of established interfaces. Reciprocal-allocation experiments show that resource exchange can respond to variation in partner contribution,^9^ ^,^ ^50^ but they do not by themselves demonstrate that plants compare individual arbuscules or selectively eliminate poorly performing fungal genotypes. Accordingly, performance-sensitive interface maintenance requires fungal contribution, differential plant response or allocation, and subsequent fate to be linked at the same interface. A claim of partner-level sanction additionally requires a measurable negative consequence for fungal growth or reproductive fitness. Table 2 provides the operational meanings and minimum evidentiary requirements used for evaluation, reward, sanction, eviction, partner choice, and related terms in this review.

**Figure 2.**
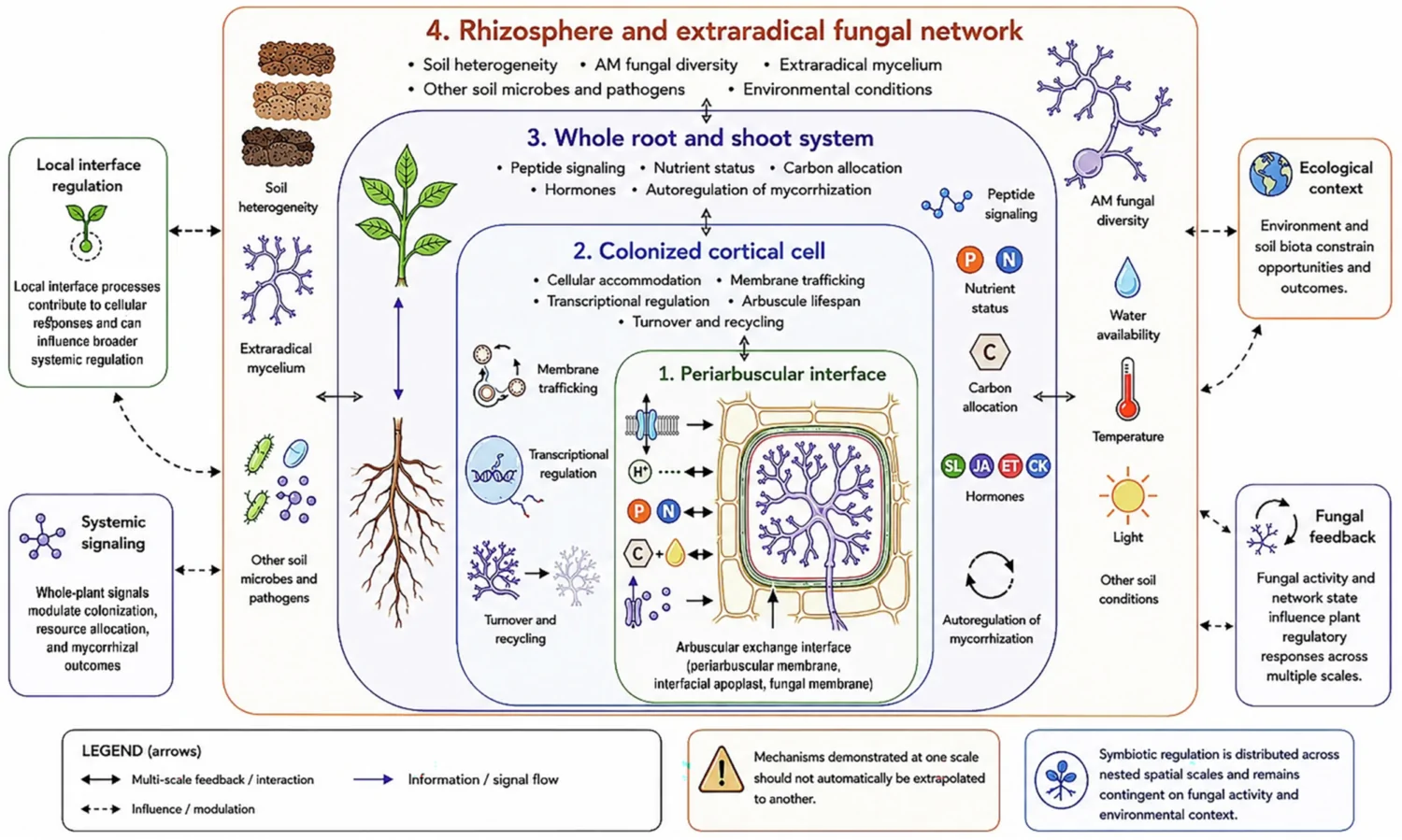
Regulation across biological scales in arbuscular mycorrhizal symbiosis. Regulatory processes operate across the periarbuscular interface, colonized cortical cell, whole root–shoot system, and rhizosphere–extraradical fungal network. Local and systemic signals, resource allocation, fungal activity, and environmental conditions interact across these nested scales. Processes demonstrated at one scale should not be assumed to operate equivalently at another.

Plant regulation also operates across biological scales. At the individual-interface or cortical-cell scale, plants regulate membrane specialization, transporter localization, nutrient uptake, and arbuscule turnover.^2^ ^,^ ^32^ ^,^ ^33^ At the root-sector and whole-plant scales, nutrient status, existing colonization, and shoot-derived signals influence subsequent colonization and resource allocation.^34-36^ At the fungal-network and ecological scales, these processes occur within heterogeneous environments and may involve multiple hosts and functionally distinct fungal partners.^39^ ^,^ ^45^ Evidence obtained at one scale should therefore not automatically be extrapolated to another. For example, altered transporter abundance in an individual arbuscule does not demonstrate preferential support of one fungal genotype across a root system.^15^ Figure 2 summarizes these spatial distinctions.

**Figure 3.**
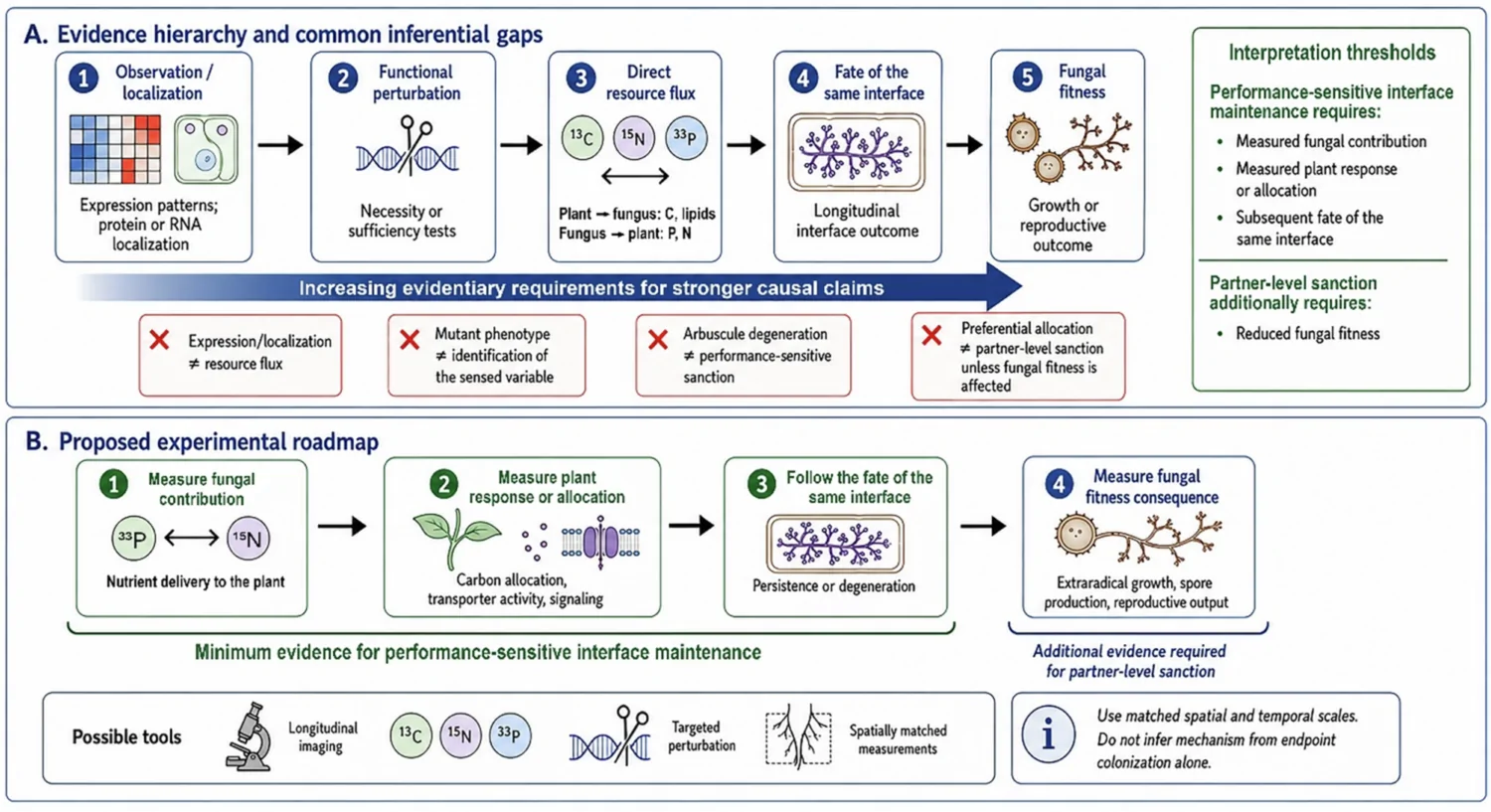
Proposed experimental roadmap for testing causal regulation in arbuscular mycorrhizal symbiosis. Panel A summarizes increasing evidentiary requirements from observation and localization to functional perturbation, direct resource flux, fate of the same interface, and fungal fitness, while highlighting common unsupported inferences. Panel B presents the authors’ proposed minimum experimental framework for testing performance-sensitive interface maintenance and partner-level sanction. Measurements should be linked at matched spatial and temporal scales.

We classify evidence by type rather than applying a formal hierarchy of study quality. Association or localization evidence identifies genes, proteins, metabolites, or signaling responses associated with AM development but does not establish causal function.^32^ Functional perturbation through mutation, silencing, chemical inhibition, or complementation provides stronger evidence when it reproducibly alters a defined symbiotic phenotype.^2^ ^,^ ^33^ Such perturbations establish functional involvement but do not necessarily identify the variable sensed or the complete mechanism. Direct resource-flux measurements establish movement of carbon or nutrients^1^ ^,^ ^50^ whereas time-resolved analysis of individual interfaces can establish how interface state relates to subsequent persistence or degeneration.^15^ In this review, claims of partner-level sanction additionally require a contribution-dependent plant response that produces a measurable consequence for fungal growth or reproductive fitness. Figure 3 presents the authors’ proposed experimental roadmap for linking these evidentiary types and should not be interpreted as a sequence already demonstrated in a single AM system.

The symbiotic phosphate transporter PT4 illustrates why these distinctions matter. Loss of PT4 function in *M. truncatula* impairs symbiotic phosphate uptake and causes premature arbuscule degeneration, demonstrating that plant nutrient-import machinery contributes to normal interface maintenance.^2^ Subsequent studies showed that nitrogen status and AMT2;3 modify the premature-degeneration phenotype of *pt4* mutants, indicating that interface lifespan is embedded within broader nutritional regulation.^30^ ^,^ ^51^ These experiments provide strong evidence for plant regulation of interface maintenance, but they do not demonstrate that PT4 itself senses fungal performance or that degeneration represents a sanction against an underperforming fungal partner. Similar caution applies to transcriptional feedback circuits and receptor kinases that regulate arbuscule development or sustained symbiosis:^12^ ^,^ ^13^ such regulators may constrain resource expenditure or maintain cellular homeostasis without directly evaluating fungal quality.

Throughout this review, claims of plant control are therefore assessed using three questions: what process is altered, at what biological scale does the alteration occur, and what evidence links the plant response to a functional symbiotic outcome? Colonization percentage, gene expression, nutrient transfer, plant growth, interface degeneration, and fungal fitness are treated as distinct readouts rather than interchangeable measures. Increased colonization does not necessarily imply increased nutrient transfer, and accelerated arbuscule degeneration does not by itself demonstrate performance-sensitive sanction of a lower-contributing fungal partner. The checkpoint framework is thus intended to separate demonstrated regulatory mechanisms from mechanistic interpretations that remain plausible but insufficiently tested, while retaining fungal agency and environmental dependence as integral components of AM symbiosis.

## 4. Invitation: Nutritional and physiological gating of fungal recruitment

Before an arbuscular mycorrhizal fungus reaches the root surface, the plant can influence whether an encounter is likely to occur through compounds that alter fungal metabolism, hyphal growth, and branching in the rhizosphere. “Invitation” therefore refers to a plant-regulated increase in the probability of fungal contact, not directed recruitment of a particular fungal individual. Most presymbiotic experiments demonstrate fungal activation or branching in response to compounds or root exudates rather than selective recruitment of a nutritionally superior partner.^10^ ^,^ ^17^ ^,^ ^52^

### 4.1. Nutrient demand establishes a permissive presymbiotic state

Phosphate availability provides the clearest evidence that fungal recruitment depends on plant nutritional status. Under phosphate limitation, plants activate a phosphate-starvation response that includes both direct nutrient-acquisition mechanisms and genes required for AM symbiosis. In rice, PHOSPHATE STARVATION RESPONSE 2 (PHR2) regulates the expression of genes involved in strigolactone biosynthesis, fungal-signal perception, common symbiosis signaling, intracellular accommodation, and symbiotic phosphate uptake. Colonization by *Rhizophagus irregularis* is strongly reduced in *phr2* mutants under low phosphate, whereas constitutive PHR2 expression partially permits colonization at phosphate concentrations that normally suppress the association. PHR2 also binds to and activates the promoter of the strigolactone-biosynthesis gene *CCD7*, and *phr2* roots release lower amounts of several strigolactones. Thus, phosphate starvation establishes a broader state of symbiotic competence rather than simply increasing production of a single fungal stimulant.^18^

Conversely, phosphate sufficiency suppresses AM development at several levels. In *M. truncatula*, high phosphate reduces fungal attachment, colonization, and expression of strigolactone-biosynthesis genes, yet roots retain nuclear calcium-spiking responses to fungal exudates and purified chitooligosaccharides. Fungi appropriately stimulated before contact nevertheless colonize high-phosphate-grown roots poorly, indicating that reduced presymbiotic fungal stimulation alone cannot explain the high-phosphate phenotype. These findings show that phosphate sufficiency can restrict progression of the interaction even when components of fungal perception remain functional, cautioning against attributing nutrient control solely to strigolactone abundance.^53^

Nitrogen status can also influence presymbiotic signaling, although responses vary among hosts. In sorghum, nitrogen and phosphorus fertilization reduce production and exudation of 5-deoxystrigol relative to nutrient-deficient conditions, whereas in red clover phosphorus deficiency, but not the other nutrient deficiencies tested, promotes orobanchol exudation. Strigolactone exudation therefore reflects host- and nutrient-dependent regulation rather than a uniform response to nutrient deficiency.^54^ ^,^ ^55^

### 4.2. Strigolactones increase encounter probability but do not guarantee entry

Strigolactones provide the strongest mechanistic example of a plant-derived presymbiotic signal. Natural strigolactones induce extensive hyphal branching in *Gigaspora margarita*, whereas studies with *Gigaspora rosea* show that strigolactones or the synthetic analog GR24 stimulate mitochondrial activity, respiration, nuclear division, and other growth-associated processes before or during enhanced fungal growth. Strigolactone release can therefore shift AM fungi toward active presymbiotic growth and increase the probability of root encounter.^10^ ^,^ ^56^ ^,^ ^57^

Genetic evidence confirms the importance of regulated strigolactone production and export. In petunia, the ABC transporter PDR1 mediates strigolactone export, and *pdr1* mutants show reduced exudation and AM colonization. In rice, mutations in the strigolactone-biosynthesis genes *D10* and *D17* strongly reduce hyphopodium formation and delay the establishment of successive colonization units, although fungal hyphae can still approach and grow around mutant roots. Strigolactones therefore promote efficient hyphopodium formation and timely entry rather than acting as indispensable long-range attractants for every colonization event.^19^ ^,^ ^20^

Natural and synthetic strigolactone structures differ in their hyphal-branching activity and effective concentrations in *G. margarita*.^58^ ^,^ ^59^ Whether such structure-dependent responses contribute to host–fungus specificity under natural conditions remains unresolved. Differential fungal responsiveness is not equivalent to plant partner choice. Demonstrating selective recruitment would require linking exudate composition to differential fungal behavior, subsequent resource exchange, and plant benefit when multiple fungal partners are simultaneously available.

Strigolactones also function in plant development and are perceived by root-parasitic plants. Evidence from early-diverging land plants supports an ancient rhizosphere-signaling role associated with fungal symbiosis, whereas their later co-option into endogenous hormonal regulation indicates that contemporary strigolactone biology integrates rhizosphere communication with plant developmental functions. Their role in invitation is therefore embedded within broader plant physiology rather than dedicated exclusively to AM recruitment.^60^

### 4.3. Light and hormonal signals regulate the invitation threshold

Presymbiotic regulation also integrates signals beyond mineral nutrition. In tomato, shoot phytochrome B signaling promotes accumulation of shoot-derived HY5 in roots, where HY5 positively regulates strigolactone-biosynthesis genes. Disruption of this pathway reduces strigolactone accumulation, AM colonization, and mycorrhiza-associated phosphate uptake under phosphate deprivation. These findings link the aerial light environment to below-ground regulation of AM establishment, although they do not demonstrate that HY5 directly senses the prospective benefit of a fungal partner.^34^

Ethylene provides an opposing regulatory input. In *Lotus japonicus*, increased ethylene signaling suppresses strigolactone-biosynthesis genes and several genes required for fungal perception and entry. Ethylene increases nuclear accumulation of SMAX1, which is required for ethylene-mediated suppression of AM development; application of strigolactone analogs or ectopic expression of CCaMK partially counteracts this suppression. The related SMAX1 pathway was previously shown in rice to repress strigolactone biosynthesis and conserved symbiotic programs downstream of D14L signaling. Ethylene therefore acts across both invitation and subsequent admission processes rather than solely by altering root exudation.^61^ ^,^ ^62^

Together, these studies indicate that the presymbiotic state integrates nutrient demand, shoot-derived signals, developmental regulation, and stress-associated hormones. The resulting invitation threshold is therefore a property of plant physiological state rather than a response to phosphate alone.^18^ ^,^ ^34^ ^,^ ^61^

### 4.4. Presymbiotic communication extends beyond strigolactones

Root exudates contain additional compounds capable of altering AM fungal behavior. In *Eustoma grandiflorum*, the monoterpene glucosides gentiopicroside and swertiamarin promote hyphal branching of *Rhizophagus irregularis* and *R. clarus*, and gentiopicroside also enhances subsequent *R. irregularis* colonization in an experimental host system. These findings show that chemically distinct metabolites can contribute to presymbiotic activation, although their broader conservation among AM hosts remains uncertain.^52^

The *N*-acetylglucosamine transporter NO PERCEPTION 1 (NOPE1) provides genetic evidence for another plant-dependent component of presymbiotic communication. Rice and maize *nope1* mutants are strongly impaired in AM establishment, and exudates from rice *nope1* roots fail to induce fungal transcriptional responses elicited by wild-type exudates. NOPE1 can transport *N*-acetylglucosamine, but the biologically relevant substrate and identity of the NOPE1-dependent signal remain unresolved. This example demonstrates that plant genotype can alter fungal presymbiotic reprogramming while illustrating why transporter activity alone cannot identify the active signal.^17^

Evidence for additional candidate presymbiotic signals should distinguish biological activity from an established recruitment function. Fungal branching or metabolic activation in vitro identifies a potentially active compound, whereas stronger evidence requires physiologically relevant production or release and an effect on subsequent symbiotic establishment in planta.^10^ ^,^ ^17^ ^,^ ^52^

### 4.5. Invitation is not equivalent to partner choice

Current evidence supports plant regulation of encounter probability through nutrient-responsive transcription, rhizosphere-signal production, transporter activity, and systemic physiological inputs. Evidence is particularly strong for phosphate-dependent regulation of strigolactone production and for strigolactone-mediated activation of fungal metabolism and branching. Invitation nevertheless remains permissive rather than decisive: stimulated fungi must still reach a susceptible root region and pass subsequent admission and accommodation checkpoints.^10^ ^,^ ^18^ ^,^ ^34^

The most defensible interpretation is therefore that plants regulate when and where fungal encounter becomes more probable, not that they select an optimal fungal partner before contact. Preferential or reciprocal allocation demonstrated after establishment of symbiosis does not itself establish presymbiotic partner choice.^9^ ^,^ ^43^ Evidence for genuine presymbiotic partner choice would require simultaneous exposure to distinguishable fungal partners and demonstration that plant-controlled signals produce differential recruitment linked to partner traits or functional contribution and subsequent symbiotic outcomes. Until then, invitation should be understood as conditional recruitment within a heterogeneous rhizosphere.

## 5. Admission: Converting fungal recognition into competence for entry

Contact with an AM fungus does not automatically result in root colonization. A fungal hypha must encounter a susceptible root region, form an entry structure, activate an appropriate host-signaling state, and induce the cellular reorganization required for penetration.^20^ ^,^ ^25^ Because chitin-derived fungal signals can engage both symbiotic and immune signaling pathways, admission cannot depend simply on detection of a uniquely AM-specific molecular signal.^21-23^ Here, admission refers to the transition from fungal perception to successful intracellular entry, conditioned by receptor composition, plant physiological state, cell identity, and downstream signaling competence.

### 5.1. Fungal signals provide overlapping rather than unambiguous identities

AM fungi produce lipochitooligosaccharides (LCOs) that stimulate symbiotic responses and can increase mycorrhizal colonization in several plant families. They also release short-chain chitooligosaccharides (COs), including CO4 and CO5, that induce nuclear-associated calcium oscillations characteristic of common symbiosis signaling.^11^ ^,^ ^63^ ^,^ ^64^ These findings, however, have not identified a single molecular signal that uniquely specifies an AM fungal partner. Fungal exudates instead contain multiple symbiotically active molecules whose relative contributions during natural colonization remain incompletely resolved.

The distinction between symbiotic and immunogenic chitin-derived signals is likewise not absolute. In *M. truncatula*, CO4–CO8 can activate nuclear calcium oscillations associated with symbiosis signaling, while CO perception can also activate immune outputs. Coordinated perception of COs and LCOs enhances symbiotic signaling and suppresses components of immune signaling. Thus, signal identity alone does not dictate outcome; concentration, receptor context, accompanying molecules, host species, and the response measured all influence interpretation.^21^

Recognition capacity also varies among hosts and physiological states. Cereal roots respond to a broad range of LCO structures, whereas legumes can show greater specificity, and nutrient starvation can increase LCO responsiveness through NSP1- and NSP2-dependent regulation of receptor expression and associated pathways. Signal availability and admission are therefore distinct: the same fungal molecule need not produce the same developmental outcome in every host or nutritional condition.^65^

The available evidence supports a combinatorial model in which plants integrate partially overlapping fungal signals rather than recognize a single unambiguous “Myc identity.” Responses to purified molecules establish signaling capacity, but they do not by themselves demonstrate which signal combinations are necessary or sufficient during colonization by a living fungus.^21^ ^,^ ^65^

### 5.2. LysM receptor complexes bias signaling toward different outcomes

Chitin-derived molecules are perceived largely through plasma-membrane LysM receptor-like kinases and receptor-like proteins. Individual LysM receptors can contribute to both immune and symbiotic responses, whereas others show greater functional specialization; admission is therefore better understood as an outcome of receptor-complex composition than as the output of a single universal AM receptor.^21-23^

In rice, OsCERK1 is required for short-chain CO perception during AM signaling and contributes to normal colonization, while also participating in chitin-triggered immunity through different receptor associations. In *M. truncatula*, CERK1 and LYR4 similarly contribute to CO-triggered symbiotic calcium oscillations and immune responses, and mutations impair AM establishment.^21^ ^,^ ^22^

More specific separation of signaling outcomes is provided by LYK8. In *M. truncatula*, LYK8 acts redundantly with CERK1 in CO-induced symbiotic signaling and colonization but is not required for the measured immune responses to CO treatment. The *lyk8 cerk1* double mutant loses CO-induced symbiotic gene activation and is strongly impaired in colonization, while LYK8 can associate with CERK1 and the common symbiosis receptor kinase DMI2. These genetic and interaction data support a role for receptor-complex composition in directing chitin perception toward symbiotic signaling.^23^

Receptor functions are nevertheless not universal across species. Comparative analyzes of LYR-IB receptors across several angiosperms, including petunia, tomato, *M. truncatula*, *Brachypodium*, rice, and wheat, reveal conserved capacities for LCO/CO recognition but differences in genetic redundancy and AM phenotypes among host systems. Variation in receptor-family composition therefore cautions against extrapolating the function of a single receptor from one host to all AM plants.^66^

Overall, LysM receptors influence whether fungal signals are routed toward symbiotic or immune outputs, but receptor activation should not itself be equated with successful entry. It establishes one component of admission that must still be integrated with downstream signaling and cellular competence.^21^ ^,^ ^23^

### 5.3. Nuclear calcium signaling establishes symbiotic competence

Downstream of surface perception, fungal signals activate the common symbiosis signaling pathway, including SYMRK/DMI2-dependent signaling, nuclear calcium oscillations, CCaMK, and CYCLOPS. Genetic analyzes in legumes and cereals demonstrate that disruption of core components of this pathway strongly impairs AM establishment.^24^ ^,^ ^64^ ^,^ ^65^

A characteristic output is repeated calcium elevation in and around the nucleus. Nuclear calcium oscillations indicate that a cell has activated symbiosis-associated signaling, but they do not demonstrate that fungal entry will occur. High phosphate can suppress colonization while preserving calcium responses to fungal signals, and some COs can activate both symbiotic and immune pathways. Calcium oscillations are therefore better interpreted as evidence of signaling competence than irreversible commitment to fungal accommodation.^21^ ^,^ ^53^

CCaMK acts as a central decoder of these calcium signals. Gain-of-function variants can activate symbiotic programs in the absence of microbial signals and induce prepenetration apparatus-like structures. In *Lotus japonicus*, constitutively active nuclear-localized CCaMK partially restores AM development in a *ccamk* mutant. This demonstrates that activation of the calcium-decoding machinery can bypass part of the upstream signaling requirement, although it does not reproduce the spatial and developmental information normally supplied by fungal contact.^24^

Activated CCaMK interacts with CYCLOPS, and the CCaMK–CYCLOPS complex subsequently cooperates with DELLA proteins to activate RAM1.^3^ These links connect early signal perception with later accommodation programs, but at the admission checkpoint their key role is to establish a cellular state permissive for intracellular progression.

Calcium-spiking patterns can vary with nutrient conditions and with the symbiotic signals examined, but the functional significance of differences in periodicity, amplitude, or frequency remains incompletely resolved.^67^ The requirement for calcium decoding is therefore much better established than the existence of a specific calcium “code” that determines admission.

### 5.4. Entry requires active preparation of the host cell

Successful penetration is preceded by extensive reorganization of the contacted plant cell. Following hyphopodium formation, the nucleus moves toward the contact site, cytoplasmic strands form, endomembrane compartments are repositioned, and cytoskeletal and secretory machinery define a transcellular route. This prepenetration apparatus forms before fungal penetration and predicts the path subsequently followed by the intracellular hypha in *M. truncatula* and karat roots.^25^ Its formation provides direct cellular evidence that entry involves active host preparation rather than simple mechanical penetration of a permissive cell wall.

CCaMK activation can induce prepenetration apparatus-like structures without a fungal partner, whereas the spatial organization of natural penetration develops in association with fungal contact. Common symbiosis signaling therefore appears to establish cellular competence, while local fungal cues contribute to the spatial context in which penetration occurs.^24^ ^,^ ^25^

VAPYRIN provides an additional link between symbiotic signaling and intracellular accommodation. In *M. truncatula*, loss of VAPYRIN reduces epidermal penetration; intercellular fungal growth can occur in the cortex, but normal arbuscule development is blocked. VAPYRIN also associates with DMI3/CCaMK, and analysis of its ankyrin-repeat domains demonstrates separable requirements for localization and intracellular accommodation functions.^26^ ^,^ ^68^ These phenotypes show that signal perception must be coupled to cellular remodeling for successful intracellular fungal development. Because VAPYRIN's precise molecular function remains unresolved, it is more appropriate to describe it as an essential component of intracellular accommodation than as a direct executor of penetration.

Host preparation should nevertheless not be interpreted as unilateral control of the entry route. AM fungi produce signals and secreted regulatory molecules that can alter host signaling and cellular responses during colonization, although their specific contributions to individual penetration events remain less well resolved.^16^ ^,^ ^40^ ^,^ ^69^ Resolving the relative plant and fungal contributions will require experiments that follow fungal contact, host signaling, cellular remodeling, and subsequent entry within the same infection event.

## 6. Accommodation: Constructing the exchange interface

Admission allows an AM fungus to enter the root cortex, but it does not ensure that the intracellular hypha will develop into a functional arbuscule. Accommodation requires extensive host-cell reorganization, including vacuolar repositioning, organelle rearrangement, and directed membrane secretion around proliferating fungal branches. Here, accommodation refers specifically to the cellular reorganization, membrane trafficking, and construction and differentiation of the intracellular interface; resource provision and establishment of exchange capacity are treated separately as investment in Section 7.

### 6.1. Accommodation is a cell-specific and asynchronous process

Colonized roots are cellular mosaics rather than uniformly mycorrhizal organs. At a given time, one cortical cell may be preparing for fungal entry, another may contain a developing arbuscule, a third may support a mature exchange interface, and an adjacent cell may be dismantling a degenerating arbuscule. Whole-root measurements therefore integrate colonized and uncolonized cells together with multiple stages of arbuscule development. Spatial co-transcriptomic analysis of *M. truncatula* roots colonized by *Rhizophagus irregularis* resolved distinct host and fungal transcriptional states associated with different stages of colonization. Arbuscule-containing regions were enriched in transcripts related to signaling, lipid metabolism, and transmembrane transport, illustrating why bulk-root expression cannot be interpreted as the state of a representative symbiotic cell.^14^

Cellular heterogeneity also has consequences for the interpretation of mutant phenotypes. A reduction in the proportion of mature arbuscules could result from defective initiation, slow branching, accelerated degeneration, or failure to replace short-lived interfaces. Similar endpoint colonization phenotypes can therefore arise from mechanistically different defects. Developmental staging and, where possible, time-resolved analysis are needed to distinguish failures of accommodation from altered arbuscule turnover.^14^ ^,^ ^16^

### 6.2. A transcriptional switch couples fungal branching to interface function

The transition from intracellular hypha to highly branched arbuscule depends on a plant transcriptional program downstream of the common symbiosis signaling pathway. Calcium decoding by CCaMK and transcriptional regulation through CYCLOPS contribute to activation of REQUIRED FOR ARBUSCULAR MYCORRHIZATION 1 (RAM1), with DELLA proteins linking symbiotic signaling to arbuscule development. Disruption of RAM1 strongly impairs normal arbuscule branching, showing that fungal admission can occur without completion of accommodation.^3^

RAM1 does not appear to operate as an isolated transcriptional regulator. In legumes, members of the WRINKLED1-like AP2 transcription-factor family, including CBX1 and WRI5 proteins, regulate genes involved in fatty-acid biosynthesis, lipid export, phosphate uptake, and arbuscule development. Recent work showed that RAM1 physically interacts with several AM-induced WRI transcription factors and that these complexes activate promoters of genes such as *RAM2*, *STR*, and *PT4*.^28^ This connects upstream symbiosis signaling with the metabolic and transport machinery required to build a functional interface.

The coordinated induction of branching, lipid metabolism, membrane trafficking, and transport machinery supports accommodation as an integrated developmental program. However, expression of PT4, RAM2, or STR indicates preparation for exchange rather than exchange itself; realized function additionally depends on protein localization and activity, substrate availability, electrochemical gradients, and fungal metabolism.^28^

Accommodation is also restrained by transcriptional feedback. In *M. truncatula*, WRI5a and the related AP2/ERF factor ERM1 activate genes involved in lipid export and arbuscule development. ERM1 and WRI5a also promote expression of ERF12, which recruits the TOPLESS corepressor and opposes the same developmental program. Disruption of this negative-feedback circuit alters arbuscule abundance and development.^12^ Thus, the accommodation program contains intrinsic negative feedback, although current evidence does not show that this circuit responds specifically to fungal nutrient delivery.

### 6.3. The host-derived periarbuscular membrane is a specialized regulatory compartment

As the arbuscule branches, the plant deposits the periarbuscular membrane closely around the fungal surface. The fungus therefore remains outside the plant cytoplasm, separated from it by the fungal plasma membrane, fungal cell wall, periarbuscular space, and plant-derived periarbuscular membrane. Arbuscule branching greatly increases potential exchange surface, but functional exchange also requires specialization of the surrounding host membrane for proton pumping, nutrient transport, carbon transfer, and turnover.^32^ ^,^ ^70^

The periarbuscular membrane is continuous with the plant plasma membrane but is not compositionally identical to it. Distinct domains form around different regions of the arbuscule. In rice, live imaging of an OsPT11–green fluorescent protein fusion localized the symbiotic phosphate transporter predominantly around fine arbuscular branches. During degeneration, rapid shrinkage of arbuscule branches precedes the subsequent loss of OsPT11–GFP signal, demonstrating dynamic turnover of the transporter-containing membrane domain during the arbuscule life cycle.^32^ Polar targeting of symbiosis-specific phosphate transporters also involves a transient reorientation of host secretion toward the developing arbuscular interface, providing further evidence that periarbuscular-membrane differentiation is actively organized by the host.^71^

Targeted secretion contributes to this membrane specialization. The exocyst subunit EXO70I accumulates near developing arbuscule branches and is required for formation of a branch-associated periarbuscular membrane domain. Mutants impaired in EXO70I function develop defective arbuscules and show abnormal localization of membrane markers.^4^ A complementary component of this secretory machinery is the symbiosis-associated syntaxin SYP132α. In *M. truncatula*, SYP132α is enriched in arbuscule-containing cells, and its knockdown prevents normal maturation of the host–fungus interface, supporting a functional requirement for specialized membrane trafficking during accommodation.^27^ These findings demonstrate an active host contribution to membrane construction, while fungal branching determines the geometry around which that membrane develops. The interface is therefore jointly shaped but asymmetrically constructed: the fungus provides the branching template, whereas the plant builds and equips much of the surrounding exchange membrane.

## 7. Investment: Resourcing the exchange interface

Investment begins once the developing interface is supplied with host-derived resources and equipped with the transport and electrochemical machinery required for reciprocal exchange. It is therefore distinct from accommodation: accommodation constructs the intracellular interface, whereas investment establishes its capacity for resource provision and nutrient recovery. Importantly, the presence or localization of this machinery establishes exchange capacity but does not by itself demonstrate the magnitude or direction of net resource flux.^1^ ^,^ ^2^ ^,^ ^72^

### 7.1. Plant carbon investment includes both carbohydrates and lipids

AM fungi depending on host-derived carbon to sustain intraradical growth, expansion of the extraradical mycelium, and spore production. Carbohydrates have long been recognized as one component of this transfer. A high-affinity fungal monosaccharide transporter, MST2, is expressed in arbuscules and other intraradical fungal structures, and its silencing impairs colonization, demonstrating that fungal sugar uptake contributes functionally to the association.^72^ MtSWEET1b is likewise induced in arbuscule-containing cells and localizes to the periarbuscular membrane. Two *Mtsweet1b* insertion mutants showed no significant AM phenotype, whereas expression of dominant-negative MtSWEET1b variants promoted premature arbuscule collapse, supporting a likely redundant contribution to glucose transport and interface maintenance rather than an indispensable single transport route.^73^ The precise routes, chemical forms, and relative quantities of carbohydrate transferred therefore remain incompletely resolved.

The discovery that plants also supply AM fungi with lipids substantially changed the model of symbiotic carbon exchange. Isotope labeling, lipid profiling, comparative genomics, and plant-mutant analyzes showed that fatty acids synthesized in colonized plant cells are transferred to *R. irregularis*. AM-specific lipid provision involves plant lipid-biosynthesis genes including *FatM* and *RAM2*, and disruption of this pathway restricts fungal development and strongly reduces transfer of plant-derived lipid to the fungus.^6^ ^,^ ^7^ RAM1–WRI transcriptional complexes additionally regulate genes including *RAM2* and *STR* 49. Although STR/STR2 are strong candidates for transfer of lipid-derived compounds across the periarbuscular membrane and are required for normal arbuscule development, their transported substrate has not been directly identified; they should therefore not be described as proven lipid exporters.^1^ ^,^ ^28^

Carbohydrate and lipid transfer should nevertheless not be treated as interchangeable components of a single carbon pool. Lipid production is tightly associated with arbuscule-containing cells and the RAM1–WRI regulatory module, whereas fungal carbohydrate acquisition can occur at several intraradical structures and may involve redundant transport pathways. Their relative contributions are therefore likely to depend on developmental and physiological context.^72^ ^,^ ^73^

Carbon availability is not determined solely within the colonized root. Competition from other plant sinks can alter fungal carbon acquisition. In split-root potato systems, competition with potato cyst nematodes altered host carbon partitioning and reduced total plant-derived carbon reaching extraradical AM fungal hyphae while maintaining fungal palmitic-acid levels; foliar aphid feeding similarly reduced fungal hexose abundance without reducing palmitic acid. This places AM carbon allocation within the wider plant source–sink economy, but does not demonstrate deliberate withdrawal of carbon from the fungus; altered photosynthesis, sink strength, transport, or host metabolism could produce the same outcome.^74^

### 7.2. Nutrient uptake gives the interface functional value

Plant investment becomes nutritionally beneficial only when fungal-acquired nutrients are recovered across the interface. Symbiosis-specific phosphate transporters provide the clearest mechanistic example. MtPT4 is located in the periarbuscular membrane of *M. truncatula* and contributes to acquisition of phosphate released by the fungus. Loss of PT4 severely impairs symbiotic phosphate uptake and causes premature arbuscule degeneration.^2^ ^,^ ^75^ This links plant nutrient-import machinery with both phosphate acquisition and normal interface persistence, but does not establish that PT4 itself senses fungal performance.

Phosphate uptake is energized by the proton gradient across the periarbuscular membrane. The AM-induced H+ -ATPase HA1 is required for efficient phosphate transport and for the positive growth response of mycorrhizal *M. truncatula*. Loss of the corresponding H+ -ATPase activity reduces proton-pumping capacity, arbuscule development, and mycorrhizal nutrient uptake, whereas OsHA1 overexpression increases membrane potential and phosphate acquisition.^29^ Thus, transporter localization alone is insufficient: uptake also depends on the electrochemical conditions established by the host.

Nitrogen is also transferred through AM associations, but transporter identity does not necessarily reveal physiological function. In *M. truncatula*, AMT2;3 is required for the nitrogen-dependent suppression of premature arbuscule degeneration in *pt4* roots. In contrast to the related AMT2;4 proteins, AMT2;3 did not restore growth of an ammonium-transporter-deficient yeast strain.^30^ These results establish a role for AMT2;3 in regulation of arbuscule lifespan but do not demonstrate conventional ammonium transport or identify the variable it may sense; a transport-related or regulatory function under specialized interface conditions remains possible.

These examples reinforce the evidence distinctions defined in Section 3: expression and localization identify candidate interface components, functional perturbation establishes biological requirement, and biochemical or tracer measurements are needed to demonstrate transport activity or net resource flux. None of these evidence types alone establishes performance-sensitive evaluation.

### 7.3. Investment creates capacity, not a guaranteed reciprocal return

Accommodation and investment establish the cellular capacity for reciprocal exchange, but a mature arbuscule does not guarantee a fixed amount of nutrient return. Realized flux depends on nutrient availability to the extraradical mycelium, fungal physiology and genotype, competition within fungal networks, plant carbon status, and transporter activities in both partners.^45^ ^,^ ^50^ ^,^ ^74^

Experiments manipulating carbon supply have shown that increased carbon availability can stimulate fungal nitrogen uptake and transfer, demonstrating reciprocal responsiveness of fungal activity to host carbon supply.^50^ Nevertheless, such responses do not reveal whether a plant evaluates the performance of individual arbuscules before allocating further carbon. Differential carbon allocation could instead reflect developmental state, local sink strength, transporter activity, or metabolic feedback.

Accommodation and investment should therefore be distinguished from evaluation. The plant clearly controls much of the machinery that constructs and resources the interface, but evidence that support is adjusted according to the nutrient contribution of an individual arbuscule remains limited. As defined in this review, demonstrating performance-sensitive interface maintenance requires fungal contribution, plant response or allocation, and subsequent fate to be linked at the same interface and at matched spatial and temporal scales. This distinction provides the transition to the next section, which considers how established arbuscules are maintained or terminated.

## 8. Maintenance and termination/renewal: What determines Arbuscule lifespan?

Arbuscule formation is not a permanent commitment. Individual arbuscules develop, mature, and degenerate over a period of days, while colonization of the root can continue through the formation of additional interfaces.^15^ ^,^ ^32^ This turnover raises a central question: is arbuscule lifespan primarily developmentally programmed, or can it be modified by nutrient exchange and plant physiological status? Current evidence supports active plant regulation of arbuscule maintenance and dismantling, but evidence that plants compare individual arbuscules and selectively terminate poorly performing ones remains limited.

### 8.1. Arbuscule lifespan is variable rather than uniform

Arbuscules are often described as short-lived structures with a standard developmental sequence from initial branching to maturity and collapse. However, recent live imaging in rice has shown substantial variation in arbuscule morphology, developmental trajectory, and lifespan. Individual arbuscules differ in branching and persistence, arguing against a strictly uniform developmental timer.^15^ ^,^ ^32^

Functional variation can occur even among arbuscules that appear morphologically similar. Live imaging of the rice symbiosis-specific phosphate transporter PT11 revealed consistent localization around fine arbuscular branches but considerable variation in transporter abundance among individual arbuscules. PT11 abundance also varied with plant phosphate conditions, showing that transporter abundance at the interface is responsive to plant phosphate demand. Because PT11 abundance was used as a proxy for symbiotic phosphate-transport capacity rather than a direct measurement of phosphate flux, morphologically similar arbuscules should not be assumed to have equivalent functional states.^15^

This heterogeneity permits the possibility of interface-level regulation but does not demonstrate performance sensing. Differences in transporter abundance or lifespan could reflect developmental age, cortical-cell state, plant nutrient demand, or fungal physiology. Demonstrating performance-sensitive evaluation requires linking nutrient delivery at a defined interface to the plant response and subsequent fate of that same arbuscule.

### 8.2. Nutrient-import capacity is connected to interface maintenance

A well-established genetic connection between symbiotic function and arbuscule lifespan comes from studies of AM-specific phosphate transporters. In *M. truncatula*, PT4 is localized to the periarbuscular membrane and is required for phosphate uptake through the mycorrhizal pathway. Loss of PT4 causes premature arbuscule degeneration, reduced intraradical fungal proliferation, and failure of the fungus to establish a sustained association. Thus, host nutrient-import machinery is required for normal interface persistence.^2^ ^,^ ^75^

Premature degeneration in *pt4* mutants is not explained simply by carbon starvation of the fungus. Degeneration still occurs when the fungus is connected to a nurse plant capable of supplying carbon, placing the defect at or near the affected host interface rather than solely at the fungal-network level. However, PT4 loss does not establish that the plant directly senses inadequate fungal phosphate delivery; altered phosphate transport or signaling, proton-coupled transport, or interface physiology could all contribute to degeneration.^51^

The *pt4* phenotype is also modified by nitrogen availability. Nitrogen deprivation suppresses premature arbuscule degeneration in *pt4* roots, and this suppression depends on the AM-induced ammonium-transporter-family protein AMT2;3. PT8, a second AM-induced phosphate transporter, is expressed at the interface under these conditions, but suppression of premature degeneration also occurs in nitrogen-starved *pt4 pt8* double mutants. PT8 compensation is therefore not required for the nitrogen-dependent rescue of arbuscule lifespan.^30^ ^,^ ^51^ These interactions indicate that interface maintenance integrates phosphorus and nitrogen status rather than monitoring one nutrient pathway in isolation, cautioning against describing premature degeneration as a simple punishment for inadequate phosphorus delivery.

The evidence therefore links nutrient-import machinery and plant nutritional state to arbuscule maintenance, while leaving unresolved whether fungal nutrient flux is itself a variable sensed by the plant.

### 8.3. Arbuscule degeneration is an active plant program

The collapse of an arbuscule might superficially resemble passive fungal senescence, but molecular evidence demonstrates that the plant activates a coordinated degeneration program. Transcriptomic comparison of wild-type and prematurely degenerating *pt4* arbuscules identified genes associated with late arbuscule development, many of which encode predicted secreted hydrolases. These include cysteine proteases, chitinases, a triacylglycerol lipase, a nuclease, and other proteins potentially involved in dismantling fungal and interface material.^33^

The symbiosis-induced transcription factor MYB1 regulates a substantial part of this program. Reducing MYB1 activity suppresses premature degeneration in *pt4* roots and decreases the expression of degeneration-associated genes, whereas constitutive MYB1 expression increases their expression and interferes with continued symbiotic development. MYB1 interacts with DELLA and NSP1, and MYB1-dependent expression of degeneration-associated genes is enhanced by DELLA and requires NSP1.^33^ These findings demonstrate active host regulation of a transcriptional program associated with interface dismantling rather than merely a response occurring after fungal collapse.

Active host dismantling does not, however, establish which partner initiates termination. The experiments defining the MYB1 program identify host mechanisms involved in degeneration but do not establish that lower fungal nutrient contribution triggers that program. “Termination” is therefore more secure mechanistically than “eviction”: the plant clearly contributes to dismantling an individual interface, but selective termination in response to poor fungal performance has not yet been demonstrated.

### 8.4. Maintenance depends on signaling at established interfaces

Regulatory constraints operate before visible degeneration. The WRI5a–ERM1–ERF12 negative-feedback circuit described in Section 6.2 restricts continued activation of arbuscule-development and lipid-associated transcriptional programs. Its physiological input remains unclear, however, and there is no evidence that the circuit specifically detects inadequate fungal nutrient return.^12^

Postdevelopmental signaling at the periarbuscular membrane may also influence whether symbiosis remains sustainable. The receptor-like kinase ARK1 is expressed in arbuscule-containing rice cells and localizes to the periarbuscular membrane. Rice *ark1* mutants can form morphologically normal arbuscules but show reduced overall colonization and substantially fewer fungal vesicles. Co-cultivation with colonized wild-type roots restores fungal vesicle and spore formation, separating initial arbuscule development from plant functions required for subsequent fungal life-cycle progression.^31^

ARK function is evolutionarily conserved. The *Marchantia paleacea* ARK protein can complement the rice *ark1* phenotype, and loss of ARK in *M. paleacea* similarly impairs sustained AM colonization, supporting an ancestral role for this kinase in symbiosis maintenance across land plants.^13^ However, the molecular input perceived by ARK and its downstream signaling mechanism remain unresolved. ARK therefore demonstrates that plant signaling associated with an established interface contributes to sustained symbiosis and fungal life-cycle completion, but not that the pathway measures fungal contribution.

### 8.5. Reward, restriction, sanction, and eviction require different evidence

Resource-allocation experiments provide evidence that AM exchange can respond to partner contribution. Experiments comparing fungal species and spatially separated fungal compartments showed preferential plant carbon allocation toward more cooperative fungal partners or fungal hyphae with access to greater phosphorus resources. Reciprocal experiments showed greater fungal phosphorus allocation toward root compartments receiving more carbon.^9^ These experiments demonstrate reciprocal plasticity in resource allocation, but their spatial scale is crucial.

Preferential carbon allocation is not equivalent to arbuscule eviction. Greater carbon supply to a productive fungal compartment or root sector does not demonstrate selective maintenance of individual arbuscules, while reduced fungal abundance could result from reduced carbon supply, suppression of new colonization, altered arbuscule renewal, or indirect growth effects. Partner-level sanction requires a contribution-dependent difference in plant treatment that produces a measurable negative consequence for fungal growth or reproductive fitness; performance-sensitive interface termination requires the contribution and subsequent fate of the same interface to be linked experimentally.

These distinctions are particularly important because one AM fungal individual can form many arbuscules across multiple cells and extraradical mycelial networks can connect different root regions or host plants.^39^ ^,^ ^48^ Local degeneration of one arbuscule therefore cannot be equated with elimination or sanction of a fungal genotype. The biological scale of the response arbuscule, cortical cell, root sector, fungal network, or fungal individual must be specified.

### 8.6. A developmental–physiological model is consistent with current evidence

As a conceptual synthesis, three alternative models can be considered for arbuscule termination. Under a fixed-timer model, arbuscule lifespan is principally programmed, and variation results from differences in developmental rate. Under a performance-sensitive model, the plant responds to variation in fungal contribution and alters interface persistence accordingly. A developmental–physiological model combines these possibilities: arbuscules follow a baseline developmental trajectory whose persistence can be modified by plant nutrient status, transporter activity, local cellular conditions, and fungal physiology.

Current evidence is consistent with the developmental–physiological model but does not establish it as a general mechanism. Variable arbuscule trajectories argue against a completely uniform timer, while transporter mutants link host nutritional physiology with premature degeneration.^2^ ^,^ ^15^ ^,^ ^30^ MYB1-dependent pathways demonstrate active plant dismantling, and ARK-dependent signaling links established interfaces with sustained colonization and fungal life-cycle progression.^13^ ^,^ ^31^ ^,^ ^33^ None of these findings alone demonstrates that lifespan is determined by the nutrient return of an individual arbuscule.

The critical missing evidence is a direct connection between performance and fate. Demonstrating performance-sensitive maintenance would require measuring fungal nutrient delivery at a defined interface, relating it to plant resource allocation or signaling in the associated cell, and then following the persistence or termination of that same arbuscule. Demonstrating a partner-level sanction would additionally require consequences for fungal fitness. Figure 3 presents the authors’ proposed minimum evidentiary chain for testing these alternatives, and Table 3 develops performance-sensitive maintenance as a testable prediction rather than an established mechanism.

**Table 3.** Testable predictions arising from the checkpoint framework for regulation of arbuscular mycorrhizal symbiosis.

Prediction	Empirical basis	Minimum discriminating experiment	Essential measurements	Result supporting the prediction	Major alternative to exclude	References
Checkpoints are genetically and experimentally separable	Genetic perturbations can impair presymbiotic recruitment, signal perception, accommodation, nutrient uptake/maintenance, or arbuscule dismantling at distinguishable stages.	For a candidate checkpoint, measure the immediately preceding transition, the checkpoint itself, and one downstream outcome; use inducible perturbation or complementation where feasible.	Measurements that bracket the affected transition, such as fungal approach/entry, calcium signaling, arbuscule development, nutrient uptake, or interface lifespan.	One transition is disrupted while preceding checkpoints remain substantially functional.	General defects in root development, nutrition, viability, or immunity.	[2,3,20,21,33]
Local and systemic regulation are experimentally distinguishable	Individual colonized cells differ in transcriptional state and arbuscule trajectory, whereas split-root studies demonstrate systemic regulation of AM development.	Use a split-root design in which one root sector receives a defined local fungal or nutrient manipulation while systemic plant status and an untreated root sector are measured. Add cell-resolved analysis only when the hypothesis concerns individual interfaces.	Local interface activity or fate; colonization in treated and untreated sectors; whole-plant nutrient status. Direct carbon or nutrient flux is required when resource allocation is the tested mechanism.	Local responses track the manipulated sector when systemic status is comparable, whereas systemic regulation produces coordinated effects in untreated sectors.	Differences in root age, cell identity, local fungal density, or developmental stage.	[14,15,36,76]
Signal dynamics may be more informative than signal abundance	AM signals induce temporally structured nuclear calcium responses, and calcium-spiking patterns vary with signal identity and nutritional context.	Follow one candidate signal and the corresponding checkpoint transition in the same cell, then perturb signal timing, amplitude, or frequency without simply abolishing the pathway.	Time-resolved signal dynamics and the probability or timing of the corresponding checkpoint transition; additional reporters are complementary rather than essential.	Defined signal dynamics reproducibly predict or alter the probability or timing of a specific checkpoint transition.	Reporter artifacts, developmental variation, or nonspecific cellular stress.	[21,63,67]
Performance-sensitive maintenance can be distinguished from programmed turnover	Nutrient-import perturbations and plant nutrient status alter arbuscule lifespan, reciprocal allocation occurs at partner or compartment scales, and individual arbuscules show heterogeneous trajectories. None of these observations links fungal contribution directly to fate of the same interface.	Create a defined difference in fungal nutrient contribution while keeping host conditions comparable; measure the plant response and follow the subsequent fate of the same connected interface.	Fungal nutrient contribution; differential plant response or allocation; persistence or degeneration of the same interface. Fungal growth or reproductive fitness is additionally required for partner-level sanction.	Performance-sensitive maintenance is supported when measured fungal contribution predicts a differential plant response and subsequent fate of the same interface. A developmental–physiological model predicts modification of, rather than complete replacement of, a baseline developmental trajectory.	Developmental age, direct effects of local nutrients on the plant cell, fungal developmental differences, or systemic changes in plant nutrient status.	[2,9,15,30]
Environmental history can be separated from current physiological state	Phosphate pulses affect developing and mature interfaces differently, and transient chitooligosaccharide treatment can produce long-lasting effects on later AM development.	Apply a prior treatment, terminate it, restore treated and control plants to comparable current physiological conditions, and then assay a defined checkpoint response.	Current physiological status and the selected checkpoint response; a persistent causal state is additionally required for a mechanistic memory claim.	A history-dependent response persists after current physiological conditions are matched; evidence for memory is stronger if perturbation of a persistent causal state removes the later phenotype.	Residual differences in nutrient stores, root architecture, plant size, water status, fungal abundance, or other current physiological variables.	[38,47]
Fungal regulatory effects are checkpoint- and partner-specific	Fungal effectors, CLE-like peptides, small RNAs, and stage-dependent secreted molecules can modify host processes, while AM fungi show substantial functional variation.	Perturb one fungal regulatory molecule in a defined host–fungus combination and measure its effect on a specified checkpoint. Rescue should be included where feasible; host or fungal genotype comparisons provide a separate test of partner specificity.	Stage-appropriate fungal-molecule expression or activity; defined checkpoint phenotype; fungal viability. A host target is required when target-specific regulation is claimed.	The perturbation changes a defined checkpoint without generally compromising fungal viability, with rescue where feasible. Reproducible differences among host–fungus combinations support partner specificity.	General loss of fungal viability or unrelated fungal developmental defects, off-target effects, or nonspecific host stress.	[16,40,42,45,69]

Note: The predictions and minimum evidentiary criteria are proposed by this review; the cited primary studies provide the empirical basis but do not necessarily test each prediction in full. The experimental designs specify the minimum comparisons needed to distinguish major alternatives. Spatial omics, multiple reporters, isotope combinations, and quantitative modeling are complementary approaches rather than mandatory components. Performance-sensitive interface maintenance requires fungal contribution, plant response or allocation, and subsequent fate to be linked at the same interface and at matched spatial and temporal scales; partner-level sanction additionally requires a measurable fungal growth or reproductive fitness consequence.

Plant control is therefore clearest in regulation of interface maintenance, dismantling, and renewal. The stronger claim that plants routinely assess and evict poorly performing fungal partners remains not yet demonstrated. In this review, “eviction” should consequently refer to regulated termination or nonrenewal of an arbuscular interface, whereas “partner-specific sanction” should be reserved for cases linking fungal performance, differential plant treatment, and fungal fitness consequences.

## 9. Local checkpoints under systemic and environmental governance

Regulation at individual arbuscules occurs within the nutritional and metabolic economy of the whole plant. A cortical cell may construct or dismantle an exchange interface locally, but resource availability and the probability of forming additional interfaces are also shaped by systemic signals. Root nutrient status, shoot carbon availability, existing colonization, and environmental conditions can therefore influence several checkpoints simultaneously. Systemic regulation integrates local symbiotic development with whole-plant nutrient demand rather than treating each fungal entry event independently.^34^ ^,^ ^36^ ^,^ ^77^

### 9.1. Autoregulation restricts further colonization

The clearest evidence for whole-plant control is autoregulation of mycorrhization, a negative-feedback process through which existing colonization and plant physiological status can restrict subsequent AM development. In *M. truncatula*, CLE–SUNN signaling provides a genetically defined component of this regulation and can alter fungal colonization and root strigolactone levels.^35^ ^,^ ^77^ Several components are homologous to machinery involved in autoregulation of nodulation, including CLE peptides, RDN1, and CLV1-like receptor kinases, but the precise mobile signal and tissue-to-tissue route of AM autoregulation remain incompletely resolved.

In *M. truncatula*, CLE53 is induced locally by AM colonization and by elevated phosphate availability. CLE53 overexpression reduces colonization in a manner dependent on the receptor kinase SUPER NUMERIC NODULES and the hydroxyproline O-arabinosyltransferase ROOT DETERMINED NODULATION1, whereas reduced CLE53 expression and mutations in SUNN or RDN1 increase colonization. Mutation of conserved proline residues in CLE53 abolishes its suppressive effect, consistent with a requirement for peptide modification. Complementary experiments showed that CLE53 and CLE33 overexpression can reduce root strigolactone levels in a SUNN-dependent manner.^35^ ^,^ ^77^

These results establish a genetic module that limits AM development, but the complete route of the signal remains unresolved. CLE53 expression near the root vasculature and its local induction adjacent to colonized cortical regions are consistent with a role in long-distance regulation, but direct movement of mature CLE53 from colonized roots to the shoot has not been demonstrated.^35^ ^,^ ^77^ Evidence that CLAVATA signaling also restricts AM colonization in tomato indicates that negative regulation is not confined to legumes, although reciprocal grafting experiments reveal receptor-specific local and shoot contributions rather than demonstrating a single conserved systemic route.^78^

Autoregulation is best distinguished from termination of existing arbuscules. Its clearest effect is to restrict further colonization, so established interfaces may persist while formation of new entry points or arbuscules is reduced. Systemic feedback can therefore modify invitation, admission, accommodation, or renewal without demonstrating selective termination of mature interfaces.^35^ ^,^ ^77^

### 9.2. Peptide signals can promote as well as suppress symbiosis

Peptide regulation is not exclusively inhibitory. Under low-phosphate conditions, several C-terminally encoded peptide genes are induced in *M. truncatula*. Overexpression of CEP1 promotes AM establishment, whereas mutants in its receptor COMPACT ROOT ARCHITECTURE 2 show reduced fungal entry, intraradical colonization, and arbuscule abundance. The *cra2* mutants also contain less strigolactone and show reduced expression of several symbiosis-associated genes. However, arbuscules that do form in *cra2* roots show no major morphological defect, indicating that the CEP–CRA2 module primarily promotes root competence and early AM establishment rather than demonstrating direct regulation of established arbuscules.^37^

The CEP–CRA2 and CLE53–SUNN modules should not be treated simply as opposing switches. They operate in different nutritional and developmental contexts and may affect different stages of symbiosis. CEP1–CRA2 signaling promotes early establishment under low-phosphate conditions, whereas CLE–SUNN signaling restricts colonization in response to colonization and phosphate-related signals.^35^ ^,^ ^37^ ^,^ ^77^ Neither pathway has been shown to measure the performance of individual fungal partners.

A second positive peptide mechanism further illustrates the diversity of CLE functions. In *M. truncatula*, CLE16 is expressed in colonized cells and promotes arbuscule abundance and persistence through the receptor-associated pseudokinase CORYNE. CLE16 signaling reduces reactive oxygen species accumulation, creating conditions more favorable for fungal development. *Rhizophagus irregularis* encodes a CLE-like peptide, RiCLE1, that also suppresses ROS accumulation and promotes colonization through MtCRN. Unlike MtCLE16, however, RiCLE1 did not measurably alter arbuscule development or abundance. These results provide direct evidence that a fungal peptide can engage a host peptide-signaling pathway as a cross-kingdom regulatory input.^16^

CLE-related signaling therefore cannot be classified as uniformly inhibitory. Different peptide–receptor modules can restrict colonization, promote AM establishment, or influence arbuscule persistence, depending on peptide origin, modification, receptor identity, and plant nutritional status.^16^ ^,^ ^35^ ^,^ ^37^

### 9.3. Shoot light and carbon status regulate below-ground investment

Because AM fungi depend on recently fixed plant carbon, shoot conditions can influence whether root colonization is supported. In tomato, shoot phytochrome B signaling promotes the accumulation of shoot-derived mobile ELONGATED HYPOCOTYL 5 (HY5) in roots, where HY5 activates strigolactone-biosynthesis genes. Disruption of this pathway reduces strigolactone accumulation, AM colonization, and mycorrhiza-associated phosphate uptake under phosphate limitation. Grafting and tissue-specific experiments support a systemic connection between shoot light perception and root symbiotic signaling.^34^

This pathway links above-ground environmental perception with the invitation checkpoint, but does not show that plants calculate the carbon cost or nutrient return of individual fungal partners. Because HY5 regulates photosynthesis, development, and nutrient acquisition, its effect on AM symbiosis may reflect coordinated changes in both carbon supply and nutrient demand.

Host carbon availability and competition from other sinks can also alter fungal resource acquisition. Increasing plant carbon availability stimulates fungal nitrogen uptake and transfer, whereas experiments in potato showed that foliar aphids and root-feeding cyst nematodes alter the quantity and chemical form of recently fixed plant carbon acquired by *R. irregularis*.^50^ ^,^ ^74^ These responses demonstrate coupling between whole-plant carbon status and fungal resource acquisition, but they do not demonstrate active withdrawal of carbon from a particular arbuscule or fungal genotype. Establishing such local regulation would require carbon transfer and interface fate to be measured at the relevant spatial scale.

### 9.4. Nutrient repletion acts differently at different checkpoints

Phosphate addition is often presented as a general inhibitor of AM symbiosis, but dynamic experiments show that different stages do not respond equally. Split-root studies in petunia and pea demonstrated that elevated phosphate can systemically suppress AM colonization or symbiotic gene expression in root compartments not directly receiving high phosphate. In pea, high phosphate also systemically suppresses strigolactone release, while exogenous strigolactone fails to restore colonization under phosphate-replete conditions. These results indicate that nutrient repletion changes both recruitment signals and downstream root competence.^36^ ^,^ ^76^

A molecular link between phosphate status and symbiotic suppression has been identified in tomato. Under phosphate-replete conditions, SlSPX1 interacts directly with several arbuscule-induced SlPHR transcription factors and represses their capacity to activate AM-associated genes. Loss of SlSPX1 enhances colonization under phosphate-replete conditions, whereas SlPHR proteins act redundantly to promote the symbiotic program.^44^ This links phosphate sensing with AM regulation without implying direct assessment of an established fungal interface.

The effect of a nutrient pulse also depends on whether an interface is developing or already mature. In colonized rice roots, phosphate treatment inhibits formation of new arbuscules within hours and arrests many developing interfaces before extensive fine branching. In contrast, preformed branched arbuscules remain structurally and metabolically intact, and their measured lifespan is not shortened. The inhibitory effect on new arbuscule development is also temporary, with new colonization resuming within approximately two days.^38^ Thus, nutrient repletion preferentially restricts formation or renewal rather than causing immediate degeneration of established arbuscules.

The effect of phosphate therefore depends on developmental state and timing. A noncolonized root may reduce recruitment, an invaded cell may restrict early arbuscule development, whereas a mature interface may persist temporarily. Nutrient repletion modifies different checkpoints with different sensitivities rather than switching the entire symbiosis off simultaneously.^36^ ^,^ ^38^ ^,^ ^76^

### 9.5. Biotic interactions can redirect systemic priorities

Existing AM colonization can alter signaling beyond the root. In rice, colonization systemically alters the expression of defense-related genes in leaves and increases resistance to the foliar blast fungus *Magnaporthe oryzae* under the experimental conditions tested.^79^ Other biotic interactions can act in the opposite direction from the plant toward its fungal symbiont by altering resource availability. In potato, foliar aphid feeding and root-feeding cyst nematodes altered the amount or form of recently fixed host carbon acquired by *R. irregularis*.^74^

The arrival of a pathogen, herbivore, nematode, or additional symbiont can therefore alter plant carbon allocation, nutrient demand, hormones, and immune signaling. Any of these changes could influence AM recruitment, investment, maintenance, or fungal growth. Yet endpoint measurements of plant biomass, pathogen abundance, or total AM colonization cannot identify which checkpoint was altered. Resolving these interactions therefore requires measurements at the developmental and spatial scale at which the regulatory response occurs.^74^ ^,^ ^79^

### 9.6. Environmental history should not yet be called symbiotic memory

Previous environmental or symbiotic experience can leave persistent physiological or molecular states, but persistence alone does not establish biological memory. General frameworks for plant priming distinguish memory from continued induction or acclimation by requiring a later altered response that persists beyond the original stimulus and cannot be explained simply by ongoing physiological differences.^46^

An AM-specific example of long-lasting history dependence comes from *M. truncatula*, where transient application of short-chain chitooligosaccharides produced effects lasting for several weeks on strigolactone biosynthesis, accommodation-related gene expression, and subsequent AM development.^47^ This demonstrates a prolonged consequence of prior signaling, but it does not by itself establish a distinct symbiotic-memory mechanism because the treatment also advances the subsequent developmental trajectory of the symbiosis.

For a claim of symbiotic memory, the prior treatment should be terminated, plants should be returned to matched current physiological conditions, and a later checkpoint response should remain dependent on the earlier experience. A persistent causal state should ideally also be identifiable and experimentally perturbable. For example, plants exposed to an earlier phosphate shortage could be returned to an equivalent nutritional state and then tested for altered strigolactone production, receptor sensitivity, carbon allocation, or arbuscule renewal. Comparable experiments could separate drought from a subsequent rewatering phase while monitoring later colonization or the fate of established interfaces. Without such matched-state designs, residual differences in nutrition, water status, plant development, established interfaces, or fungal abundance cannot be distinguished from memory.^46^ “History dependence” is therefore more appropriate than “symbiotic memory” until a previous condition can be shown to alter a later response after the original physiological differences have been experimentally removed.^46^ ^,^ ^47^

Systemic governance is best understood as cross-scale coordination rather than a single centralized regulatory pathway. Peptide signaling, phosphate status, shoot light perception, carbon availability, and biotic interactions can modify recruitment, accommodation, investment, maintenance, and renewal in a stage-dependent manner.^16^ ^,^ ^34^ ^,^ ^38^ Importantly, systemic regulation of these checkpoints does not demonstrate that individual arbuscules or fungal genotypes are evaluated according to performance. The fungal partner can in turn alter its own resource allocation and provide regulatory molecules that engage host signaling pathways.^16^ ^,^ ^39^ AM regulation is therefore asymmetrical but reciprocal, with local interface processes embedded within whole-plant physiology and fungal-network activity.

## 10. Fungal regulatory inputs as cross-scale modifiers of plant control

The checkpoint framework emphasizes plant regulation of fungal recruitment, entry, accommodation, investment, maintenance, and termination. These processes are not controlled by the plant alone. AM fungi modifying host signaling, adjust their metabolism to host-derived resources, and redistribute nutrients through extraradical networks that extend beyond individual colonized cells.^39^ ^,^ ^40^ ^,^ ^50^ Fungal regulatory inputs therefore modify plant regulation at interface, root, whole-plant, and network scales.

Evidence for fungal regulatory effects varies in strength because AM fungi are obligate biotrophs and remain difficult to manipulate genetically. Ectopic expression or host-induced gene silencing can identify candidate functions, whereas stronger mechanistic evidence additionally requires secretion or transfer during symbiosis, localization in the relevant host compartment, interaction with a host target, and a functional effect on colonization or exchange.

### 10.1. Fungal effectors modify host cellular programs

SECRETED PROTEIN 7 (SP7) provided early evidence that *Rhizophagus irregularis* actively modifies host regulation. SP7 contains a secretion signal, localizes to the plant nucleus when expressed in plant cells, and interacts with the defense-associated transcription factor ERF19. Constitutive expression of SP7 in *M. truncatula* roots increases fungal colonization.^40^ These findings support a role in modifying host defense-related transcription, although much of the mechanistic evidence derives from ectopic expression rather than direct quantification of native SP7 delivery during colonization.

SP7 belongs to a broader family of fungal proteins capable of modifying host RNA regulation. SP7-like effectors from different AM fungi localize to plant nuclear condensates and interact with components of the mRNA-processing machinery. When expressed ectopically in plants, these effectors alter alternative splicing of a subset of host transcripts.^80^ This extends potential fungal influence beyond individual defense pathways to host RNA processing, although the extent to which these effects occur in naturally colonized cortical cells remains to be established.

RiNLE1 provides evidence for modification of host chromatin-associated processes. It is highly expressed during arbuscule development, localizes to the plant nucleus, and interacts with histone H2B. Ectopic expression of RiNLE1 reduces H2B monoubiquitination and defense-associated gene expression and increases mycorrhizal colonization. Attempts to reduce RiNLE1 expression by host-induced gene silencing were unsuccessful, so a fungal loss-of-function requirement for RiNLE1 has not been demonstrated.^41^ Together, these results support the capacity of fungal effectors to alter transcriptional, chromatin-associated, or post-transcriptional states in host cells, while also illustrating that the strength of functional evidence differs among individual effectors.^40^ ^,^ ^41^ ^,^ ^80^

### 10.2. Fungal proteins can modify the molecular environment encountered by plant receptors

Fungal influence also occurs outside the plant cytoplasm. The *R. irregularis* LysM protein RiSLM is a putatively secreted candidate effector that binds chitin and chitosan in vitro. Its expression is reduced during nonfunctional interactions with *mtpt4-2* and *vapyrin* hosts, and RiSLM homologs among *R. irregularis* isolates show substantial sequence diversity.^81^ These findings identify RiSLM as a candidate for modifying chitin-derived molecular signals, but they do not by themselves demonstrate suppression of plant immunity, protection of fungal hyphae from plant chitinases, or a requirement for colonization.

This distinction is particularly relevant at the admission checkpoint. Plant LysM receptor complexes contribute to the balance between symbiotic and immune signaling responses to chitin-derived molecules,^21-23^ whereas fungal chitin-binding proteins could potentially modify the accessibility of these ligands to host receptors. Schmitz et al.^81^ identified RiSLM as a short, putatively secreted LysM protein from *Rhizophagus irregularis* that binds chitin and chitosan in vitro and exhibits substantial sequence diversity among fungal isolates. However, the study did not establish an in planta role for RiSLM in suppressing host immunity or promoting AM colonization.^81^

### 10.3. Fungal peptides can engage host regulatory pathways

Fungal regulatory inputs can also involve molecular mimicry of plant signals. The *R. irregularis* CLE-like peptide RiCLE1 has functional similarities to plant CLE signaling, and RiCLE1 overexpression or peptide application increases root colonization. Its symbiosis-promoting effect depends on the host pseudokinase CORYNE and is associated with reduced root reactive oxygen species accumulation.^16^ Unlike the related plant peptide MtCLE16, however, RiCLE1 does not measurably increase arbuscule abundance, maturation, or lifespan.

The evidence does not show that RiCLE1 can override an otherwise nonpermissive host or that the mechanism is conserved across AM fungi. Its native processing and secretion and direct interaction with a cognate receptor during natural colonization remain to be resolved. RiCLE1 is therefore best interpreted as evidence that regulatory pathways normally classified as components of host control can also receive fungal inputs.^16^

### 10.4. Cross-kingdom small RNAs provide another regulatory route

Recent evidence shows that *R. irregularis* small RNAs can enter plant cells and associate with *Lotus japonicus* AGO1 during colonization. Several predicted host targets can be cleaved by cognate fungal small RNAs in vitro, and sequestration of four candidates fungal small RNAs in transformed host roots reduces fungal colonization.^42^

Cross-kingdom RNA transfer therefore adds a post-transcriptional mechanism through which fungi can alter host regulatory state. However, its prevalence across fungal lineages, hosts, developmental stages, and symbiotic checkpoints is not yet established. The identities and functions of many host targets also remain unresolved, so broader roles in immunity, trafficking, metabolism, or accommodation should remain hypotheses unless specific fungal RNAs, host targets, and cellular phenotypes are linked experimentally.^42^

### 10.5. Fungal regulatory output depends on host and developmental context

Fungal signaling is not expressed as a fixed program. Transcriptomic analysis of the *R. irregularis* secretome shows differences among extraradical hyphae, intraradical structures, and arbuscule-containing cells, as well as variation among host plants.^69^ Fungal regulatory output therefore depends on both developmental stage and host context.

This context dependence limits generalization from individual host–fungus combinations. Mechanisms demonstrated for *R. irregularis* in *M. truncatula*, for example, cannot automatically be assumed to operate identically in rice, tomato, or other hosts. Host genetic background can itself contribute substantially to variation in mycorrhizal responsiveness, as demonstrated by genetic analyzes of AM responses in maize.^82^ Independent studies also demonstrate substantial functional and genetic variation among AM fungal isolates or genotypes in extraradical growth, phosphorus acquisition and delivery, effects on plant growth, and responses to host identity or phosphate conditions.^45^ ^,^ ^83^ ^,^ ^84^ Observed host–fungus specificity may therefore arise at different checkpoints and should not be interpreted as specific recognition or partner choice unless the relevant mechanism is identified.

Host and fungal responses may additionally form feedback loops. A plant nutrient or signaling state can alter fungal gene expression, after which fungal molecules modify the plant response.^40^ ^,^ ^50^ ^,^ ^69^ In such cases, assigning a regulatory event exclusively to one partner obscures the reciprocal nature of the mechanism.

### 10.6. Fungal regulation extends to resource allocation at the network scale

At the fungal-network scale, AM fungi actively acquire, transport, store, and release nutrients rather than functioning as passive conduits controlled by plant transporters. Increased host carbon availability can stimulate fungal nitrogen acquisition and transfer and alter fungal nitrogen-metabolism gene expression.^50^ In common mycorrhizal networks, fungi preferentially allocate phosphorus and nitrogen toward hosts with greater carbon-source strength, while lower-carbon hosts can remain colonized and continue receiving fungal nutrients.^39^

Reciprocal-allocation experiments similarly show greater fungal phosphorus allocation toward root compartments providing more carbon, while plants can allocate more carbon toward fungal partners associated with greater phosphorus supply.^9^ These results establish resource-responsive allocation by both partners, although they do not identify the mechanism responsible for the allocation differences; local resource concentrations, transporter responses, sink strength, and network architecture remain possible contributors.

The scale distinction is critical. A fungal individual may connect multiple root sectors or host plants while forming many arbuscules.^39^ Nutrient allocation across that network therefore cannot be inferred solely from events at one cortical interface, just as degeneration of one arbuscule cannot be interpreted as elimination of the entire fungus.

Natural roots may also contain multiple AM fungi and interact simultaneously with other symbionts, pathogens, herbivores, and soil microorganisms. Plant responses to AM fungi can differ strongly among fungal species and isolates.^83^ ^,^ ^85^ Under mixed conditions, changes in carbon or nutrient allocation can therefore reflect competition among multiple plant and fungal sinks rather than an isolated bilateral response. Tests of partner-specific control require fungal identity to be resolved within mixed colonization and linked to resource flux or interface fate.

### 10.7. Plant regulation is substantial but not unilateral

Fungal regulatory inputs do not negate plant regulation. Plants exert substantial control over cellular entry and interface construction and provide the carbon, lipids, and transport machinery required to establish and sustain intracellular exchange.^1^ ^,^ ^2^ ^,^ ^4^ Plant nutritional and systemic signaling can also restrict new arbuscule development or subsequent colonization without necessarily terminating established interfaces.^35^ ^,^ ^38^

The fungus, however, influences many of the same transitions. Nuclear effectors can modify host transcription, chromatin, and RNA processing ^40^ ^,^ ^41^ ^,^ ^80^; fungal peptides and small RNAs can enter host regulatory pathways ^16^ ^,^ ^42^; and the extraradical mycelium regulates nutrient acquisition and distribution in response to host resource availability.^39^ ^,^ ^50^ Plant checkpoints should therefore be viewed as regulatory transitions that are strongly host governed but continuously modified by fungal activity.

We consequently favor a model of asymmetrical, reciprocal regulation rather than unilateral plant control. The plant has particular leverage over cellular access and carbon investment, whereas the fungus determines much of its soil exploration and nutrient acquisition and can regulate nutrient allocation while altering host cellular states. Neither partner determines symbiotic outcome independently.

This distinction also defines the limits of the “invitation-to-eviction” metaphor. Plants can increase encounter probability, permit intracellular progression, resource an interface, and regulate its maintenance or termination, but fungi influence each of these processes. “Eviction” is therefore retained only as an operational shorthand for regulated termination or nonrenewal of a local arbuscular interface; it does not imply cognition, punishment, or removal of the connected fungal individual or genotype.

## 11. A predictive research agenda for testing the checkpoint framework

The checkpoint framework will be useful only if it generates predictions that can distinguish among competing explanations of AM development. Many current experiments measure colonization, transcript abundance, or plant biomass at a single endpoint. These measurements identify potentially important responses but cannot determine whether a plant process acts during fungal recruitment, entry, interface construction, resource exchange, or arbuscule termination. Future studies should therefore resolve when and where a perturbation acts and connect molecular responses to resource flux and interface fate. The goal is not simply to identify additional symbiosis-associated genes, but to test whether defined signals alter the probability or timing of specific checkpoint transitions. The six predictions below are proposed tests of the framework rather than mechanisms assumed to be established. Table 3 summarizes the minimum experiments needed to distinguish competing explanations, whereas Figure 3 presents the authors’ proposed evidentiary roadmap.

### 11.1. Prediction 1: The checkpoints are genetically and experimentally separable

The framework predicts that invitation, admission, accommodation, investment, maintenance, and termination and renewal can be at least partly dissociated genetically or experimentally. For example, reduced fungal stimulation need not imply defective intracellular signaling, and successful fungal entry need not guarantee normal arbuscule construction or maintenance.^2^ ^,^ ^3^ ^,^ ^17^

For a specific checkpoint, the minimum discriminating experiment should measure an immediately preceding transition, the candidate checkpoint itself, and a relevant downstream outcome in the same genotype or treatment. A complete assay of every stage from root exudation to interface degeneration is informative but is not required for every question. Stage-specific complementation or inducible perturbation would further distinguish direct checkpoint functions from secondary effects of reduced colonization. Strong evidence for separability would be a perturbation that alters one transition while preceding checkpoints remain substantially intact.

### 11.2. Prediction 2: Local and systemic regulation are experimentally distinguishable

A central proposition of the framework is that local interface regulation and systemic whole-plant governance can be experimentally distinguished, even though the two may interact. Spatially resolved studies reveal heterogeneity among colonized cells, whereas systemic and split-root experiments demonstrate that signals generated in one part of the plant can alter AM development elsewhere.^14^ ^,^ ^35^ ^,^ ^36^ Testing this proposition requires preserving spatial scale: whole-root transcript abundance or total colonization cannot reveal whether a cortical cell responds to its local interface or to systemic plant status.

Spatial co-transcriptomics already allows plant and fungal transcriptional states to be mapped within colonized roots, while recent non-invasive imaging has captured complete arbuscule development and collapse together with changes in the rice phosphate transporter PT11. These approaches provide the necessary foundation for relating local interface state to systemic regulation.^14^ ^,^ ^15^

The minimum experiment is a split-root design in which a defined local condition is altered in one root sector while another sector remains untreated, with systemic plant status and the relevant checkpoint outcome measured in both compartments.^35^ ^,^ ^36^ ^,^ ^76^ Cell-specific reporters or spatial profiling can then be added when interface-level resolution is required. Different root sectors could encounter contrasting nutrient conditions or fungal genotypes while systemic nutrient status is controlled. If local conditions dominate interface maintenance, otherwise comparable arbuscules should differ according to the nutrient environment or physiology of the fungal structures connected to them. If systemic regulation dominates, interfaces in separate root sectors should respond similarly to signals generated elsewhere in the plant. Intermediate outcomes would indicate interaction between local and systemic inputs.

### 11.3. Prediction 3: Signal dynamics may be more informative than signal abundance

Most molecular studies determine whether a signaling pathway is activated, but the checkpoint model predicts that the duration, frequency, amplitude, and cellular location of activation may influence whether colonization proceeds. Calcium signaling provides a tractable example: AM fungal signals induce nuclear calcium oscillations, and spiking patterns vary with symbiotic signal and phosphate context, although a causal calcium “code” that determines admission has not been established.^63^ ^,^ ^67^

The minimum test should follow one candidate signal and the corresponding checkpoint transition in the same cell over time. Perturbations that alter signal amplitude, duration, or periodicity without simply abolishing the pathway would provide a stronger causal test. Simultaneous measurement of additional variables such as ROS, membrane trafficking, periarbuscular pH, transporter recruitment, or fungal growth would be valuable but should be considered optional extensions rather than minimum requirements. Causality would be supported if a reproducible change in signal pattern altered the probability or timing of a defined checkpoint transition.

This prediction also guards against the assumption that stronger signaling necessarily promotes symbiosis. Excessive or prolonged activity may trigger negative feedback, cellular stress, or termination; the relevant parameter may therefore be a signaling window rather than maximal pathway activation.

### 11.4. Prediction 4: Performance-sensitive maintenance can be distinguished from programmed turnover

The most consequential unresolved question is whether plants adjust the lifespan of individual arbuscules according to the resources delivered through them. Current evidence links host nutrient-import machinery to arbuscule maintenance and demonstrates reciprocal resource allocation at partner or compartment scales, but neither establishes that the nutrient contribution of an individual interface determines its subsequent fate.^2^ ^,^ ^9^ ^,^ ^30^

The minimum discriminating experiment must link three measurements at the same interface and at matched times: (i) fungal nutrient contribution, (ii) a differential plant response or resource allocation, and (iii) the subsequent persistence or termination of that interface. Nutrient access of the connected extraradical fungus should be experimentally manipulated while whole-plant nutritional status is kept as comparable as possible. Stable-isotope tracing, transporter or metabolic reporters, and longitudinal imaging are possible approaches for obtaining these measurements, but no particular technology is itself a requirement for demonstrating performance-sensitive maintenance. Live tracking of individual arbuscules provides a foundation for such experiments.^15^

Three outcomes would distinguish competing models. Under a predominantly programmed model, arbuscule lifespan would remain similar despite variation in measured nutrient delivery. Under a performance-sensitive model, variation in fungal contribution would predict a differential plant response or allocation and the subsequent fate of the same interface. Under a developmental–physiological model, nutrient delivery or plant nutritional state would modify, but not fully determine, a baseline developmental trajectory. Evidence for partner-level sanction would require an additional step: differential interface treatment must produce a measurable consequence for the growth or reproductive fitness of the connected fungus. Figure 3 distinguishes these evidentiary requirements explicitly.

### 11.5. Prediction 5: Environmental history can be separated from current physiological state

Most studies compare continuously treated and untreated plants. Such designs cannot determine whether earlier nutritional or environmental experience changes a later symbiotic response. The checkpoint model predicts that previous phosphate limitation, drought, shading, pathogen attack, or AM colonization could alter subsequent checkpoint responses, but this must be distinguished from persistent physiological differences caused by the original treatment. This distinction follows the broader experimental separation between persistent physiological responses and biological memory.^46^

An AM-specific precedent for history dependence comes from transient chitooligosaccharide treatment of *M. truncatula*, which produces effects lasting for several weeks on strigolactone biosynthesis, accommodation-related transcription, and subsequent AM development. These results demonstrate a prolonged consequence of previous signaling but do not by themselves establish a distinct molecular-memory mechanism.^47^ History-dependent experiments should separate prior exposure from current physiological state. For example, plants could experience phosphate deprivation and then be returned to an equivalent phosphate status before fungal inoculation. Strigolactone release, fungal activation, calcium responsiveness, entry frequency, and arbuscule development could then be compared with plants lacking the earlier treatment. Similar designs could apply drought and rewatering either before or after fungal colonization.

A persistent response alone would not demonstrate molecular memory. Residual differences in root architecture, stored nutrients, plant size, or fungal abundance must first be excluded. Within the operational definition used here, stronger evidence would require a later checkpoint response under matched current conditions and, ideally, identification of a persistent causal state whose perturbation removes that history-dependent phenotype.^46^

### 11.6. Prediction 6: Fungal regulatory effects are checkpoint- and partner-specific

Fungal regulatory inputs now documented in AM symbiosis include CLE-like peptide signaling, nuclear effectors, and cross-kingdom small-RNA transfer. These findings establish several routes through which fungal molecules can modify host regulatory processes, although the evidentiary strength differs among individual molecules. Cross-kingdom RNA interference, for example, has been functionally demonstrated in a defined *Rhizophagus irregularis–Lotus japonicus* association.^16^ ^,^ ^40^ ^,^ ^42^

The framework predicts that a fungal molecule should be assigned to a particular checkpoint only when perturbation of that molecule changes a defined developmental transition. The minimum experiment should therefore perturb one fungal regulatory molecule in a defined host–fungus combination and measure the immediately preceding state, the predicted checkpoint outcome, and the relevant host response. A host target should be validated when target-specific regulation is claimed, whereas rescue experiments are valuable where technically feasible. This avoids describing a molecule simply as “promoting colonization” when its developmental site of action is unresolved.

Comparisons across fungal isolates and host genotypes are equally important. A mechanism demonstrated in one plant–fungus combination should not automatically be generalized to AM symbiosis as a whole. Substantial genomic variation among *R. irregularis* isolates, host- and stage-dependent expression of fungal secreted proteins, and functional diversity among AM fungi provide independent reasons to test conservation explicitly.^45^ ^,^ ^69^ ^,^ ^86^ Conservation, host dependence, and fungal-genotype dependence should therefore be tested separately.

### 11.7. From descriptive phenotypes to causal tests

Across all six predictions, the minimum informative experiment is not a maximal multi-omics program. It should use a defined plant–fungus combination under a measured nutritional and environmental context, apply a perturbation relevant to one question, and measure the smallest set of outcomes needed to distinguish the competing explanations. Colonization percentage should be accompanied, where relevant, by developmental staging, plant and fungal biomass, carbon and nutrient flux, or fungal reproduction according to the hypothesis being tested rather than requiring all of these measurements in every experiment. Fungal fitness measurements become essential specifically when partner-level sanction is claimed.

No single technology is required to demonstrate plant regulation. Live imaging, isotope tracing, spatial transcriptomics, cell-specific perturbation, fungal genotyping, and quantitative modeling should instead be treated as optional tools selected according to the biological question.^14^ ^,^ ^15^ Crucially, when a causal claim depends on linking variables especially fungal contribution, plant response, and interface fate the relevant measurements should be connected within the same biological unit and at matched spatial and temporal scales rather than obtained from separate experiments and inferred to form a causal sequence.

The central experimental challenge is therefore to move from correlation to prospective prediction. A mechanistic model should specify which signal changes, at which checkpoint and spatial scale, what downstream transition should follow, and what result would falsify that interpretation. Such experiments would allow the checkpoint framework to distinguish developmental programming, metabolic feedback, systemic regulation, and performance-sensitive responses rather than grouping them under the broad label of “plant control.” Table 3 summarizes these minimum discriminating experiments and separates them from optional enabling technologies.

## 12. Conclusions

Arbuscular mycorrhizal symbiosis is not established through a single act of fungal recognition followed by an automatically sustained mutualism. Instead, plants regulate six overlapping checkpoints invitation, admission, accommodation, investment, maintenance, and termination and renewal by modulating fungal recruitment and entry, constructing and equipping the periarbuscular interface, providing carbon and lipids, recovering fungal-delivered nutrients, and regulating interface persistence and dismantling. AM development is therefore better understood as a series of overlapping and revisable checkpoints than as an irreversible linear sequence.

The strength of evidence for plant control differs among these checkpoints. Plant regulation of strigolactone production and common symbiosis signaling is supported by genetic and physiological evidence, while interface construction and active arbuscule dismantling are supported by defined host trafficking and transcriptional mechanisms. Much weaker evidence supports the stronger claim that plants directly compare the performance of individual interfaces or fungal partners and selectively terminate those providing lower returns. Reciprocal resource-allocation experiments and time-resolved analyzes of individual arbuscules provide important foundations for testing this possibility, but they do not establish performance-sensitive termination at the same interface. Arbuscule turnover should therefore not be equated automatically with partner sanction, nor preferential resource allocation with eviction of a fungal individual.

Plant control is also neither absolute nor exclusively plant driven. AM fungi providing regulatory inputs through secreted nuclear proteins, CLE-like peptides, and cross-kingdom small RNAs that can alter host regulatory processes. Fungal nutrient acquisition and allocation also respond to host carbon availability and operate through extraradical networks extending beyond individual interfaces. The association is consequently governed by asymmetrical, reciprocal regulation: the plant controls access to living cortical cells and much of the machinery that constructs and resources the intracellular interface, whereas the fungus influences host signaling and regulates its own growth, nutrient acquisition, and nutrient allocation.

The six-checkpoint framework, with “invitation to eviction” retained only as an operational shorthand rather than implying cognition or intentional partner judgment, provides a testable structure for integrating these processes across cellular, whole-plant, and ecological scales. Its value will depend on experiments that connect signaling, resource flux, interface fate, and fungal fitness at matched spatial and temporal scales. In particular, performance-sensitive interface maintenance requires fungal contribution, differential plant response or allocation, and subsequent fate to be linked at the same interface, whereas a claim of partner-level sanction additionally requires a measurable consequence for fungal growth or reproductive fitness. Such tests are needed to distinguish developmental programming and metabolic feedback from genuinely performance-sensitive regulation, and ultimately to define both the extent and the limits of plant control over AM symbiosis.

## Data Availability

No new data was generated for this study.
